# The management of surgical patients in the emergency setting during COVID-19 pandemic: the WSES position paper

**DOI:** 10.1186/s13017-021-00349-0

**Published:** 2021-03-22

**Authors:** Belinda De Simone, Elie Chouillard, Massimo Sartelli, Walter L. Biffl, Salomone Di Saverio, Ernest E. Moore, Yoram Kluger, Fikri M. Abu-Zidan, Luca Ansaloni, Federico Coccolini, Ari Leppänemi, Andrew B. Peitzmann, Leonardo Pagani, Gustavo P. Fraga, Ciro Paolillo, Edoardo Picetti, Massimo Valentino, Emmanouil Pikoulis, Gian Luca Baiocchi, Fausto Catena

**Affiliations:** 1 Service de Chirurgie Générale, Digestive, Metabolique, Centre Hospitalier de Poissy/Saint Germain en Laye, Poissy, France; 2 Department of General Surgery, Macerata’s Hospital, Macerata, Italy; 3 Department of Trauma and Acute Care Surgery, Scripps Memorial Hospital, La Jolla, CA USA; 4 Department of General Surgery, University Hospital of Varese, University of Insubria, Varese, Italy; 5 Trauma Surgery, Denver Health, Denver, CO USA; 6 Department of Emergency and Trauma Surgery, Rambam Health Campus, Haifa, Israel; 7 Department of Surgery, College of Medicine and Health Sciences, UAE University, Al-Ain, United Arab Emirates; 8 Department of Emergency and Trauma Surgery, Bufalini Hospital, Cesena, Italy; 9 Department of Surgery, University of Pisa, Pisa, Italy; 10 Abdominal Center, University Hospital Meilahti, Helsinki, Finland; 11 University of Pittsburgh School of Medicine, F-1281, UPMC-Presbyterian, Pittsburgh, PA 15213 USA; 12 Infectious Diseases Unit, Bolzano Central Hospital, Bolzano, Italy; 13 Departamento de Cirurgia, Faculdade de Ciências Médicas (FCM) –Unicamp, Campinas, SP Brazil; 14 Spedali Civili di Brescia, ASST degli Spedali Civili di Brescia, 25123 Brescia, Italy; 15 Department of Anesthesia and Intensive Care, Parma University Hospital, Parma, Italy; 16 Department of Radiology, Tolmezzo Hospital, Tolmezzo, Italy; 17 Department of Surgery, Attikon General Hospital, National & Kapodistrian University of Athens (NKUA), Athens, Greece; 18 Department of Surgery, University of Brescia, Brescia, Italy; 19 Department of Emergency and Trauma Surgery, Parma University Hospital, Parma, Italy

**Keywords:** COVID-19, Emergency surgery, Personal protective equipment, 2019-nCoV, SARS-CoV-2, Laparoscopy, Open surgery, Trauma surgery, Non operative management, Screening, Follow-up, Postoperative care, Pandemic

## Abstract

**Background:**

Since the COVID-19 pandemic has occurred, nations showed their unpreparedness to deal with a mass casualty incident of this proportion and severity, which resulted in a tremendous number of deaths even among healthcare workers. The World Society of Emergency Surgery conceived this position paper with the purpose of providing evidence-based recommendations for the management of emergency surgical patients under COVID-19 pandemic for the safety of the patient and healthcare workers.

**Method:**

A systematic review was conducted in accordance with the Preferred Reporting Items for Systematic Review and Meta-analysis Protocols (PRISMA-P) through the MEDLINE (PubMed), Embase and SCOPUS databases. Synthesis of evidence, statements and recommendations were developed in accordance with the GRADE methodology.

**Results:**

Given the limitation of the evidence, the current document represents an effort to join selected high-quality articles and experts’ opinion.

**Conclusions:**

The aim of this position paper is to provide an exhaustive guidelines to perform emergency surgery in a safe and protected environment for surgical patients and for healthcare workers under COVID-19 and to offer the best management of COVID-19 patients needing for an emergency surgical treatment.

We recommend screening for COVID-19 infection at the emergency department all acute surgical patients who are waiting for hospital admission and urgent surgery. The screening work-up provides a RT-PCR nasopharyngeal swab test and a baseline (non-contrast) chest CT or a chest X-ray or a lungs US, depending on skills and availability. If the COVID-19 screening is not completed we recommend keeping the patient in isolation until RT-PCR swab test result is not available, and to manage him/she such as an overt COVID patient.

The management of COVID-19 surgical patients is multidisciplinary.

If an immediate surgical procedure is mandatory, whether laparoscopic or via open approach, we recommend doing every effort to protect the operating room staff for the safety of the patient.

## Background

An incomparable outbreak of respiratory illness in Wuhan, Hubei Province, China, was detected in December 2019. A novel coronavirus was identified on 12 January 2020 and called the 2019 novel coronavirus (2019-nCoV); on 11 February 2020, the World Health Organization (WHO) officially indicated this viral disease, which affects mostly the lower respiratory tract and manifests as pneumonia in humans, as COVID-19.

COVID-19 has rapidly spread and the WHO declared it a Public Health Emergency of International Concern with a risk assessment of very high at a global level.

Since the COVID-19 pandemic has occurred, nations showed their unpreparedness to deal with a mass casualty incident of this proportion and severity: hospital leadership and individual providers faced difficult decisions about how to conserve critical resources, such as hospital and intensive care unit (ICU) beds, respirators, transfusion capacity, and personal protective equipment (PPE) that are vital for protecting patients and staff from unnecessary exposure and intra-hospital transmission. It resulted in high mortality rate of infected patients, in particular of fragile patients such as people with multiple chronic comorbidities and polypharmacy, and an unacceptable number of infected healthcare workers and deaths.

Since the early phase of the pandemic, surgical theatres were converted into additional ICUs to support critically ill patients and non-urgent, non-cancer surgical procedures were cancelled or postponed until a later date; all nurses and medical staff including residents were reallocated in COVID units.

In this apocalyptic scenario, the emergency surgeons accepted their crucial role in the management of infected and non infected patients and the need to work safely to limit the spread of the virus in healthcare facilities and to decrease morbidity and mortality rate, which may result from delay diagnosis and treatment of surgical patients.

The World Society of Emergency Surgery educational board (WSES) conceived this position paper with the purpose of providing recommendations for the management of surgical patients in emergency setting under COVID-19 pandemic for the safety of the patient and healthcare workers based on available evidences and experienced surgeons’ opinion.

## Method

The scientific board of WSES established 11 PICO (Patient, Intervention, Comparator, Outcomes) questions organized into 4 topics (diagnosis, preoperative management, surgical management; postoperative management) to build the position paper structure about the management of confirmed, uncertain and negative surgical patients during the COVID-19 pandemic, summarized in Table 1.

**Table 1:** Summary of statements and recommendations

**1-What is the diagnostic work-up in a suspected COVID-19 patient with an acute surgical condition?**	
**Statement 1.1**	
Symptoms of COVID-19 infection are myriad and may include stroke or myocarditis as the first presentation. COVID-19 infection is suspected in patients presenting with fever, cough, dyspnoea and/or recent direct contact with a confirmed COVID-19 patients (QoE moderate B).	
**Statement 1.2**	
Characteristic laboratory findings for COVID-19 infection are leucopenia, lymphocytopenia, elevated aspartate aminotrasferase, inflammatory biomarkers such as C-reactive protein, erythrocyte sedimentation rate; elevated lactate dehydrogenase; creatinine; hypersensitive troponin I, fibrinogen and D-dimer (QoE moderate B).	
**Statement 1.3**	
The RT‐PCR test in respiratory samples (swab) is the current gold standard method for confirming the diagnosis of COVID‐19 (QoE moderate B).	
**Statement 1.4**	
The RT-PCR test result heavily relies on the presence of viral genome in sufficient amounts at the site of sample collection that can be amplified. An incorrect sample collection or missing the time-window of viral replication can provide false negative results and limits the usefulness of qPCR-based assay (QoE moderate B).	
**Statement 1.5**	
In the early stage of the disease the detection of SARS‐CoV‐2 viral RNA is better in nasopharynx samples than the oropharynx (QoE moderate B).	
**Statement 1.6**	
For individuals with a high clinical suspicion of SARS-CoV-2 infection with negative RT-PCR test, a combination of repeated naso-pharyngeal RT-PCR swab tests and chest imaging may be helpful to confirm early the diagnosis of COVID-19 disease and to evaluate the pneumonia’s severity (QoE moderate B).	
**Statement 1.7**	
In the COVID-19 screening, the chest-CT scan is the most accurate radiological tool to confirm the diagnosis above all in uncertain cases. The chest-XR can be helpful in case of unavailability of CT-scan (QoE moderate B).	
**Statement 1.8**	
The chest-CT scan may be useful to complete the COVID-19 screening in patients with a high clinical suspicion of SARS-CoV-2 infection but negative RT-PCR swab test (QoE moderate B).	
**Statement 1.9**	
For emergency physicians and emergency surgeons with excellent POCUS skills and limited access to CT, it is reasonable to use lung POCUS in COVID-19 screening, that can help in the diagnosis and at the same time rules out other acute respiratory illnesses (QoE low C).	
**Statement 1.10**	
Lungs US can be used as first COVID-19 screening tool and discriminate low-risk patients (lung US-negative, clinically stable patients that can wait for second level imaging) from higher-risk patients (such as those with abnormal lung US patterns), that might require second level imaging rapidly (QoE very low D).	
**Statement 1.11**	
Lungs US may be helpful for patients with a high clinical suspicion of COVID-19 but negative RT-PCR test to confirm the diagnosis, if they demonstrate typical lung ultrasound findings for COVID-19, if skills are available, in the unavailability of CT-scan (QoE moderate B).	
**Recommendations/1**	
**We recommend screening for COVID-19 infection at emergency department, all surgical patients with clinical and epidemiologic features suspect for COVID-19 disease who are waiting for hospital admission and urgent surgery. The screening provides performing a RT-PCR naso-pharyngeal swab test and a baseline (non-contrast) chest CT or chest X-ray or lungs US, depending on skills and availability (Strong recommendation based on moderate level of evidence 1B).**	
**2-Is it necessary to delay the surgical procedure for a suspected COVID-19 patient until RT-PCR swab test result is available?**	
**Statement 2.1**	
All acute surgical patients should complete preoperative COVID-19 screening that includes RT-PCR naso-pharyngeal swab test and chest CT scan, when it’s available, or a Chest XR, or Lungs US in ED, whether they are symptomatic or not, to control the in-hospital spreading of SARS-CoV-2 (QoE moderate B).	
**Statement 2.2**	
Chest imaging such as a baseline CT scan or a Chest XR or a lungs US, depending on the availability, are useful diagnostic tool in the unavailability of RT-PCR swab test result to detect potentially infected patients (QoE moderate B).	
**Statement 2.3**	
If chest radiological evaluation by CXR, or chest CT scan or lungs US, is inconclusive and the patient needs for immediate surgery, he has to be treated as a COVID-19 patient to limit the risk of contagion and the spreading of the SARS-CoV-2 in the operating theatres (QoE moderate B).	
**Statement 2.4**	
After surgery, the uncertain patient has to be isolated as long as the RT-PCR test result is obtained, to be admitted in a COVID (+) or (-) ward. If it is positive, it is recommended repeating the swab test for confirmation. In patients with confirmed COVID-19 diagnosis, the laboratory evaluation should be repeated to evaluate for viral clearance prior to being released from isolation (QoE moderate B).	
**Statement 2.5**	
TACS classification system could be a valid tool to evaluate timing of surgery and severity of the surgical disease (QoE low C).	
**Recommendations/2**	
**We recommend completing the COVID-19 screening (RT-PCR nasopharyngeal swab test + chest imaging) for all acute surgical patients before admission in the surgical ward or operating room. If the RT-PCR swab test result is not available to confirm the diagnosis, the patient needs to be isolated and treated such as COVID-19 (+) patients with all the mandatory precautions. The acute care surgeon is the only responsible for the decision of possible delaying of a surgical procedure in the emergency setting during the pandemic. TACS classification is a good tool to evaluate timing of surgery. According to this classification, surgery cannot be postponed for class1 (immediate surgery) and class 2 (surgery in 1 hour, as soon as possible) patients even if diagnosis of COVID-19 is not yet confirmed by RT-PCR swab test (Strong recommendation based on a moderate level evidence 1B).**	
**3-In case of RT-PCR test unavailability and negative Chest CT Scan, suspected COVID-19 patients have to be operated using operating theatres’ procedures for overt COVID-19 patients?**	
**Statement 3.1**	
RT-PCR test remains the reference standard to make a definitive diagnosis of COVID-19 infection and to manage the patient and resources in the correct way (QoE moderate B).	
**Statement 3.2**	
The emergency physician may identify high risk COVID-19 patients investigating the presence of typical clinical symptoms, laboratory test results and/or epidemiological risk factors as suggested byWHO, but RT-PCR test confirmation is mandatory to make diagnosis of viral infection (QoE moderate B).	
**Statement 3.3**	
Negative chest CT scan is not sufficient to exclude the diagnosis of COVID-19 infection, above all in the early phase of the infection (QoE low C).	
**Statement 3.4**	
In case of unavailability of the RT-PCR test, the surgical patient has to be considered potentially infected and managed like a COVID-19 (+) patient (QoE moderate B).	
**Recommendations/3**	
**If it is not possible to confirm diagnosis of COVID-19 disease in an acute surgical patient by RT-PCR swab test, we recommend managing the patient such as he/she is COVID-19 (+) with all the mandatory precautions against viral infection, that include all the protective measures and a dedicated pathway for the operating room, to decrease the risk of environmental contamination and health personnel exposure. If a dedicated pathway for COVID-19 (+) patients is not available in the hospital, it should be an option to transfer hemodynamic stable suspected patient to the nearest COVID-19 HUB hospital for the appropriate management (Strong recommendation based on a moderate level of evidence 1B).**	
**4-In case of RT-PCR swab test unavailability and chest CT scan unavailability, suspected COVID-19 surgical patients have to be operated using operating room procedure for overt COVID-19 patient?**	
**Statement 4.1**	
Diagnosis of COVID 19 disease is confirmed through the RT-PCR test (QoE moderate B)	
**Statement 4.2**	
Each surgical patient might be considered suspected for COVID-19 disease if clinical signs, imaging features at CXR or/and lungs US or/and chest CT scan and laboratory tests results are compatible with a SARS-CoV-2 infection (QoE moderate B).	
**Statement 4.3**	
The COVID-19 screening includes RT-PCR swab test and a chest radiological imaging that could be CXR or lungs US in the unavailability of CT scan. (QoE moderate B).	
**Statement 4.4**	
If a surgical patient cannot complete the screening for COVID-19 disease, and requires immediate surgical procedure, he/she should be managed with all the mandatory precautions against COVID-19 infection (QoE low C).	
**Statement 4.5**	
If the RT-PCR swab test is positive, the surgical patient has to be manage such as a COVID-19 patient. The chest imaging is useful to assess the severity of the pneumonia (QoE high A).	
**Recommendations/4**	
**In case of RT-PCR test and chest CT scan unavailability, we recommend completing the COVID-19 screening with Chest XR or lungs US that can help assessing the severity of COVID-19 pneumonia, exactly such as chest CT scan, before surgery. If the naso-pharyngeal swab test is positive, the patient is a COVID-19 confirmed patient (Strong recommendation based on moderate level of evidence 1B).**	
**5. Are emergency surgery indications for a confirmed COVID-19 patient different?**	
**Statement 5.1**	
Indications for a surgical procedure are not different in confirmed COVID-19 patients. (QoE moderate B).	
**Statement 5.2**	
Current data about outcome of surgery in COVID-19 has shown a higher morbidity and mortality rate in comparison with negative patients (QoE moderate B).	
**Statement 5.3**	
The risk of environmental contamination and virus exposure in operating room related to the surgical management of a confirmed COVID-19 patient is high in the lack of trained health staff and personal protective equipments (QoE moderate B).	
**Statement 5.4**	
During COVID-19 pandemic, it is fundamental to carefully evaluate case by case the necessity for immediate surgical or non operative strategies, as recommended in international guidelines (QoE moderate B).	
**Recommendations/5**	
**In evaluating the necessity to perform emergency surgery in COVID-19 (+), we recommend complying with international guidelines about immediate surgery or non operative strategies, evaluating case by case and resources. According to TACS classification, class 1 and 2 patients require surgical treatment in a very short delay (Strong recommendation based on a moderate level of evidence 1B).**	
**6-Are emergency surgical procedures for confirmed COVID-19 patients different?**	
**Statement 6.1**	
SARS-CoV-2 is presumed to spread primarily via respiratory droplets and aerosols and close contact, but the virus can be isolated also in the faeces and biological fluids of the infected patient (QoE high A).	
**Statement 6.2**	
Human coronaviruses can persist on inanimate surfaces such as metal, glass, or plastic for up to nine days (QoE high A).	
**Statement 6.3**	
Aerosol generated procedures (AGP) are considered responsible for the dissemination of the SARS-CoV-2 virus in the hospital (QoE moderate B).	
**Statement 6.4**	
Performing or being exposed to a tracheal intubation without adequate PPE is the main risk factor for health care workers SARS-CoV-2 infection (QoE high A).	
**Statement 6.5**	
Laparoscopic approach has been advocated such as a high risk AGP because of the artificial pneumoperitoneum and smoke generated from the surgical devices (QoE very low D).	
**Statement 6.6**	
Laparotomy such as laparoscopy should be considered a high risk procedure that can be implicated in the intra-hospital dissemination of the virus because of the higher exposure to biological fluids, surgical smoke generated with the use of electrocautery (QoE low C).	
**Statement 6.7**	
The laparoscopic approach could have the advantage of decreasing the length of hospital stay of an asymptomatic COVID-19 patient and the risk of in-hospital infection of a negative patient, in a period of limited availability of beds (QoE very low D).	
**Statement 6.8**	
The emergency surgeon has the responsibility to evaluate if a safe surgical procedure is possible considering the restricted access to resources and the safety of surgical staff and of patient (QoE high A).	
**Recommendations/6**	
**If an immediate surgical procedure needs to be performed, whether laparoscopic or via open approach, we recommend doing every efforts to protect the operating room staff, in the safety of the patient (Strong recommendation based on low level evidence 1C). To perform a safe surgical procedure, we recommend having a trained staff, wearing the necessary PPEs and an established protocol for the preoperative, peri-operative and postoperative management of the COVID-19 surgical patient (Strong recommendation based on low level evidence 1C).We recommend being careful in the establishment and management of the artificial pneumoperitoneum, in the management of the hemostasis and of incisions to prevent any loss of biological fluids and contamination of the surgical staff (Strong recommendation based on low level evidence 1C).We recommend using of all available devices to remove smoke and aerosol during a laparoscopic procedure and a closed suction system for artificial pneumoperitoneum (Strong recommendation based on a low level evidence 1C).If it is not possible to perform surgery in a safe and protected environment, we recommend do not underestimating the highest risk of contamination and infection for health care workers and dissemination of the virus in the hospital and to consider transferring hemodynamically stable patients in a COVID HUB hospital for the appropriate management (Strong recommendation based on a low level evidence 1C).We recommend to not be present during the intubation and extubation maneuvers, if it is possible (Strong recommendation based on a moderate level evidence 1B).**	
**7-Confirmed COVID-19 patients have a different Low Molecular Weight Heparine (LMWH) prophylaxis ?**	
**Statement 7.1**	
COVID-2019 infection can activate coagulation cascade through various mechanisms, leading to severe hypercoagulability. Early anticoagulation may block clotting formation and reduce microthrombus, thereby reducing the risk of major organ damages (QoE moderate B).	
**Statement 7.2**	
In confirmed COVID-19 patients, routine D-dimer testing on admission and serially during hospital stay should be considered to stratify the risk of venous thromboembolism (VTE). In the case of significantly elevated D-dimer levels (≥1.5–2.0 mg/L), pharmacological VTE prophylaxis should be initiated (QoE moderate B).	
**Statement 7.3**	
Prophylactic-dose LMWH should be initiated in all surgical patients with COVID-19 disease admitted to the hospital to decrease thromboembolic risk related to the infection and emergency surgery (QoE moderate B).	
**Statement 7.4**	
Prophylactic anticoagulation reduces the risk of VTE in acutely ill hospitalized medical patients when the risk of bleeding is acceptable (QoE moderate B).	
**Statement 7.5**	
Anticoagulant therapy mainly with LMWH appears to be associated with better prognosis in severe COVID‐19 patients, according to the risk of surgical bleeding (QoE moderate B).	
**Statement 7.6**	
If pharmacological VTE prophylaxis is indicated, LMWH should be given at a dosage approved for high-risk situations. In case of contraindications for anticoagulation, physical measures should be used (e.g., medical compression stockings) (QoE moderate B).	
**Statement 7.7**	
Intensified VTE prophylaxis (e.g. with an intermediate, half-therapeutic LMWH dosage once daily or with a high-risk prophylactic LMWH dosages twice daily) should be considered in patients with additional risk factors (e.g. body mass index > 30 kg/m2, history of VTE, known thrombophilia, active cancer) or requiring ICU admission or with rapidly increasing D-dimer levels, taking into account renal function and bleeding risk (QoE moderate B).	
**Statement 7.8**	
Following discharge from hospital, prolonged pharmacological VTE prophylaxis is reasonable in patients with persistent immobility, high inflammatory activity, and/or additional risk factors (QoE low C)	
**Statement 7.9**	
In hospitalized COVID-19 patients who develop VTE, especially in those requiring ICU admission, LMWH at therapeutic dosages may be considered the standard of care. In cases of severe renal insufficiency, unfractionated heparin should be administered (QoE moderate B).	
**Recommendations/7**	
**We recommend administering prophylactic anticoagulation with LMWH as soon as possible in COVID-19 surgical patients to reduce thromboembolic risk related to the virus, sepsis and emergency surgery. The dosage of the anticoagulant therapy has to be adjusted according to the risk of surgical bleeding, renal function and weight of the patient (Strong recommendation based on a moderate level evidence 1B).If it is not possible to administer an antitromboembolic prophylaxis, think to the intermittent pneumatic compression, in case of immobilized patient, and to mobilize the patient as soon as possible (Strong recommendation based on moderate level of evidence 1B).**	
**8-Is postoperative treatment for confirmed COVID-19 patients different?**	
**Statement 8.1**	
COVID-19 surgical patient requires a multidisciplinary approach, above all if he/she is admitted in ICU for mechanical ventilation and presents with signs of septic shock (low level of evidence C).	
**Statement 8.2**	
Specific pharmacological treatment for COVID-19 disease is not available but when an empirical treatment is administered, it is mandatory to monitor for early detection of complications (moderate level of evidence B).	
**Statement 8.3**	
Currently there are no data about the use of antimicrobial in COVID-19 patients to prevent secondary health-care infections (low level of evidence C).	
**Statement 8.4**	
Initial prompt antibiotic therapy for intra-abdominal infections in surgical patients is typically empirical and depends on the underlying severity of infection, the pathogens presumed to be involved, and the risk factors indicative of major resistance patterns. Antimicrobial treatment should be targeted to results from cultures from the site of infections or hemocultures with de-escalation of treatment as early as possible, in according with WSES guidelines (moderate level of evidence B).	
**Statement 8.5**	
Empirical antifungal treatment should only be considered in critically COVID-19 patients, presenting fever of unknown origin, with new pulmonary infiltrate superimposed on a viral pneumonitis pattern, with the aim of confirming the diagnosis by invasive techniques and/or the use of fungal biomarkers (moderate level of evidence B).	
**Recommendations/8**	
**We recommend carefully administering antibiotics in COVID-19 surgical patients for the high risk of selecting resistant bacteria, especially in patients admitted in ICU for mechanical ventilation. Early empirical antibiotic treatment should be targeted to results from cultures, with de-escalation of treatment as soon as possible (Strong recommendation based on a moderate level of evidence 1B).**	
**9-Is it necessary to create an overt COVID-19 patient surgical ward?**	
**Statement 9.1**	
Patients needing for a surgical procedure or undergone urgent surgery with confirmed SARS-CoV-2 infection require a multidisciplinary approach and management and they need to be isolated from negative patients to decrease the in-hospital risk of virus transmission and environmental contamination (very low level of evidence D).	
**Statement 9.2**	
Confirmed COVID-19 patients need to be cared by a trained and skilled workforce with adequate PPEs (e.g N95 masks, goggles, double gloves, face mask and protective gowns) to preserve negative surgical patients from contagion, because the high risk to come in contact with droplets and biological fluids (very low level of evidence D).	
**Recommendations/9**	
**After an emergency surgical procedure, we recommend re-admitting in Covid-ICU patients with severe pneumonia for management and monitoring.For stable asymptomatic or mild symptomatic COVID-19 patients, it would be better to create a surgical dedicated ward with the aim to avoid any contamination of negative patients and to limit the in-hospital exposure to the virus to a dedicated and trained team (Strong recommendation based on a very low quality evidence 1D).**	
**10-Is it necessary to create a suspected COVID-19 patient surgical ward ?**	
**Statement 10.1**	
Insufficient precaution in managing a false negative COVID-19 patient could cause the contagion of nurses, surgeons and negative patients (QoE moderate B).	
**Recommendations/10**	
**Considering the high infectivity related to SARS-CoV-2, we suggest that suspected/uncertain patients should be isolated to ensure the limiting of exposure and contagion. If suspected/uncertain COVID-19 patient needs to undergo immediate surgery, he/she has to be managed like a confirmed COVID-19 patient, till diagnosis is confirmed by RT-PCR test, that has to be performed twice in uncertain patients.If the swab test is negative, but CT scan showed signs of COVID-19 pneumonia, the patient can’t be considered COVID-19 (-) and the RT-PCR swab test has to be repeated; if RT-PCR is positive, the patient is considered COVID-19 (+) and addressed to Covid-ICU or Covid-Surgical unit (Strong recommendation based on low level of evidence 1C).**	
**11-Are there different discharge policies for suspected/overt Covid patients?**	
**Statement 11.1**	
Current data reported that several patients meeting criteria for hospital discharge, could show positive RT-PCR test after the established quarantine of 14 days (low level of evidence C).	
**Statement 11.2**	
There aren’t data proving the contagiosity of a recovered patient who keeps to intermittently eliminate SARS-CoV-2 after 14 days from the onset of symptoms or positive RT-PCR test.	
**Recommendations/11**	
**We suggest that after hospital discharge, all the confirmed surgical COVID-19 patients should be kept in isolation for at least 2 weeks have passed since the date of their first positive naso-pharingeal swab test and until negative RT-PCR nasofaringeal swab test is obtained (Weak recommendation based on very low quality of evidence 2D).**	

A systematic review was conducted in accordance with the Preferred Reporting Items for Systematic Review and Meta-analysis Protocols (PRISMA-P) [1] through the MEDLINE (PubMed), Embase and SCOPUS databases. Every effort was made to provide a comprehensive assessment of the published evidence. The following keywords and/or Medical Subject Headings were used: “COVID-19” or “pandemic” or “pneumonia” or “2019-nCoV” AND “surgery” or “abdominal pain”, “laparoscopy”, “emergency”, “open abdomen”, “outcome”, “SARS-CoV-2” and “postoperative care” or “contamination” or “dissemination”. All available articles (reviews, editorials, epidemiological studies, case series and research letters) on COVID-19 and surgery published in the English language between 15 December 2019 and 15 July 2020 were included in the review.

A group of experts were designed by a steering committee to review the articles included in the analysis.

Synthesis of evidence, statements and recommendations were developed in accordance with the GRADE methodology [2].

The first draft of the paper was submitted to the steering group, consisting of emergency and trauma surgeons, anesthesiologists, emergency physicians, radiologists, infection disease physicians and qualified nursing personnel, for evaluation and approval.

Given the limitation of the evidence, the current document represents an effort to provide consensus-based general guidelines for surgical patient management during COVID-19 pandemic in an emergency setting.

## Results

### What is the diagnostic work-up in a suspected COVID-19 patient with an acute surgical condition?

#### Statement 1.1

Symptoms of COVID-19 infection are myriad and may include stroke or myocarditis as the first presentation. COVID-19 infection is suspected in patients presenting with fever, cough, dyspnoea and/or recent direct contact with a confirmed COVID-19 patient (QoE moderate B).

#### Statement 1.2

Characteristic laboratory findings for COVID-19 infection are leucopenia, lymphocytopenia, elevated aspartate aminotransferase, and inflammatory biomarkers such as C-reactive protein, erythrocyte sedimentation rate, elevated lactate dehydrogenase, creatinine, hypersensitive troponin I, fibrinogen and d-dimer (QoE moderate B).

#### Statement 1.3

The RT-PCR test in respiratory samples (swab) is the current gold standard method for confirming the diagnosis of COVID-19 (QoE moderate B).

#### Statement 1.4

The RT-PCR test result heavily relies on the presence of viral genome in sufficient amounts at the site of sample collection that can be amplified. An incorrect sample collection or missing the time-window of viral replication can provide false-negative results and limits the usefulness of qPCR-based assay (QoE moderate B).

#### Statement 1.5

In the early stage of the disease the detection of SARS-CoV-2, viral RNA is better in nasopharynx samples than the oropharynx (QoE moderate B).

#### Statement 1.6

For individuals with a high clinical suspicion of SARS-CoV-2 infection with negative RT-PCR test, a combination of repeated nasopharyngeal RT-PCR swab tests and chest imaging may be helpful to confirm early the diagnosis of COVID-19 disease and to evaluate the pneumonia’s severity (QoE moderate B).

#### Statement 1.7

In the COVID-19 screening, the chest CT scan is the most accurate radiological tool to confirm the diagnosis above all in uncertain cases. The CXR can be helpful in case of unavailability of CT scan (QoE moderate B).

#### Statement 1.8

The chest CT scan may be useful to complete the COVID-19 screening in patients with a high clinical suspicion of SARS-CoV-2 infection but negative RT-PCR swab test (QoE moderate B).

#### Statement 1.9

For emergency physicians and emergency surgeons with excellent point-of-care ultrasound (POCUS) skills and limited access to CT, it is reasonable to use lung POCUS in COVID-19 screening, which can help in the diagnosis and at the same time rules out other acute respiratory illnesses (QoE low C).

#### Statement 1.10

Lungs US can be used as the first COVID-19 screening tool and discriminate low-risk patients (lung US-negative, clinically stable patients that can wait for second-level imaging) from higher-risk patients (such as those with abnormal lung US patterns), which might require second-level imaging rapidly (QoE very low D).

#### Statement 1.11

Lungs US may be helpful for patients with a high clinical suspicion of COVID-19 but negative RT-PCR test to confirm the diagnosis if they demonstrate typical lung ultrasound findings for COVID-19 and if skills are available, in the unavailability of CT scan (QoE moderate B).

#### Recommendations 1

We recommend screening for COVID-19 infection at the emergency department and of all surgical patients with clinical and epidemiological features suspected for COVID-19 disease who are waiting for hospital admission and urgent surgery. The screening provides performing a RT-PCR nasopharyngeal swab test and a baseline (non-contrast) chest CT or chest X-ray or lungs US, depending on skills and availability (strong recommendation based on the moderate level of evidence 1B).

#### Summary of evidence and discussion

Patients presenting with acute abdominal pain are admitted in the emergency department (ED) for surgical evaluation, appropriate laboratory tests and abdominal imaging, if they are required to make diagnosis.

There is not yet recommendation about the routinely testing for COVID-19 disease of patients admitted in the ED with acute abdominal pain.

Since the COVID-19 infection outbreak, the WHO and CDC [3, 4] have recommended to perform diagnostic investigations for COVID-19 on the basis of a clinical judgement, in symptomatic patients, presenting in the ED with fever, nonproductive cough, dyspnoea, myalgia and fatigue, not taking in consideration the evidence of an unknown group of asymptomatic patients (or with atypical clinical signs) that can carry the virus and could transmit the infection [5].

.In clinical practice, this recommendation turned out rapidly in a large number of infected healthcare workers and a high risk of environmental contamination, worsening the problem of limited resources in terms of PPE availability, hospital beds shortage in ICU and overloaded healthcare personnel.

At ED admission, a patient is defined “suspected” for COVID-19 infection if:

- He presents with fever and at least one sign/symptom of respiratory disease and a history of travel to or residence in a country area or territory reporting local transmission of COVID-19 disease during the14 days prior to symptom onset;
- He presents with any acute respiratory illness, having been in contact with a confirmed COVID-19 case in the last 14 days prior onset of symptoms;
- He presents with severe respiratory infection, with no other etiology that fully explains the clinical presentation, requiring hospitalization.

Many authors reported that the most common symptoms in patients with confirmed COVID-19 infection are fever > 38.5 °C, breathing difficulties and dry cough, diarrhoea and vomiting are rare [6, 7].

Characteristic laboratory findings in COVID-19 pneumonia are leucopenia and lymphopenia. The level of aspartate aminotransferase is often elevated. Infected patients could present with myocarditis and an increased level of hypersensitive troponin I [6, 7]. Inflammatory biomarkers such as C-reactive protein, erythrocyte sedimentation rate and pro-inflammatory cytokines are usually elevated, as are lactate dehydrogenase, creatinine and prothrombin time [6, 7]. Prothrombin time is only minimally prolonged. The more common coagulation findings with COVID-19 are hypercoagulability evident in uniquely elevated fibrinogen and d-dimers [8, 9].

Nevertheless, several patients, even with a COVID-19 infection, do not complain of these symptoms and require admission in a surgical ward for early management of an acute abdomen.

According to the policy of infection control, during this pandemic, COVID-19 disease diagnosis should be prioritized in case of patients admitted for a surgical intra-abdominal disease to comply with the objectives of:

- Minimizing the exposure in operating room (OR);
- Decreasing the risk of environmental contamination;
- Minimizing the occupation of the OR;
- Reducing the hospital stay of patients submitted to surgery.

Suspected clinical diagnosis of COVID-19 is confirmed through [10–12]:

- The *COVID-19 RT-PCR test* that provides nucleic acid detection in the nasal and throat swab sampling or other respiratory tract samplings by real-time quantitative polymerase chain reaction (RT-PCR) and further confirmed by high-throughput sequencing [10].
- The *chest imaging* that includes chest radiograph, computed tomography (CT) scan or lungs ultrasound (US).

The application of high-throughput sequencing technology in clinical diagnosis is limited because of its equipment dependency and high cost; consequently, the RT-PCR test is the most common, effective and straightforward method for detecting pathogenic viruses in respiratory secretions.

The RT-PCR method heavily relies on the presence of viral genome in sufficient amounts at the site of sample collection that can be amplified. If the swab test result is positive, it is recommended that the test is repeated for confirmation [3]. In patients with confirmed COVID-19 diagnosis, the laboratory evaluation should be repeated to evaluate for viral clearance prior to being released from isolation. False-negative results have been observed related to the quality of the kit, the collected sample or performance of the test [11].

To limit the number of false-negative patients, the WHO has suggested collecting specimens from both the upper respiratory tract (nasal- and oropharyngeal samples) and lower respiratory tract such as expectorated sputum, endotracheal aspirate or bronchoalveolar lavage (BAL) [3].

COVID-19 patients have different virus loads or positive rates in different stages of the disease and in different parts of the body. A high viral load can be detected in the early stage of the disease by pharyngeal swab, and detection of SARS-CoV-2 viral RNA is better in nasopharynx samples than the oropharynx. In the middle stage, the viral load of the lower respiratory tract will be significantly higher than that of the upper respiratory tract. According to the existing evidence, the positive rate from high to low is bronchoalveolar lavage fluid, sputum, nasal swab, fibrobronchoscope brush biopsy, pharyngeal swab and faeces. The positive rate of nasal swabs is close to that of sputum. It is important to note that with the recovery of the disease, the positive rate of oropharyngeal swabs in mild patients declines the fastest, and in the later course of the disease, positive results of anal swabs are more than that of pharyngeal swabs. The viral nucleic acids in the stool of the recovered patients turn to negative later than the oropharyngeal swabs [13].

Furthermore, missing the time-window of viral replication can increase false-negative patients.

At present, the specificity of RT-PCR viral swab for COVID-19 is thought to be high while the sensitivity is likely < 90%. The detection of SARS-CoV2 using RT-PCR can achieve a sensitivity of 50–79%, depending on the protocol used, the sample type and the number of clinical specimens collected [12].

Therefore, while testing can miss COVID-19-infected patients as a result of the relatively poor sensitivity of the PCR viral swab, most of these false-negative patients will have a clinical picture or radiological chest feature that is consistent with the SARS-CoV-2 infection and will be presumed to have the diagnosis even with a negative swab result. This group of patients represents the uncertain COVID-19 patients to consider in the risk of in-hospital transmission of the virus.

The chest CT examination has demonstrated a high sensitivity in the initial diagnosis of the novel coronavirus pneumonia and to evaluate the severity of the infection [14, 15].

Yoon et al. [16] reported that patients with COVID-19 pneumonia show largely bilateral lesions that are patchy and also confluent, appearing as ground-glass or with a mixed consolidative and ground-glass pattern. The lesions often have a wedge-like appearance with a pleural base. Major consolidations may show air bronchograms. Pleural effusion is absent. Patchy or confluent lesions tend to be distributed along the pleura. The lobe most frequently affected is the lower right lobe, followed by the upper and lower left lobes. The posterior lung is involved in 67% of studied cases.

Another study showed that pleural effusion is present in about 32% [17].

Shi et al. [14] demonstrated that COVID-19 pneumonia manifests with chest CT imaging abnormalities, even in asymptomatic patients, with rapid evolution from focal unilateral to diffuse bilateral ground-glass opacities that progressed to or co-existed with consolidations within 1–3 weeks. Combining assessment of imaging features with clinical and laboratory findings could facilitate early diagnosis of COVID-19 disease.

Multiple patchy ground-glass consolidation (in severe pneumonia), crazy-paving pattern, interlobular thickening, adjacent pleura thickening and linear opacities in bilateral multiple lobular with periphery distribution are typical chest CT imaging features of the COVID-19 pneumonia [14, 15, 18].

A recent study [19] showed that the sensitivity of chest CT was greater than that of RT-qPCR (98% vs. 71%, respectively, *p* < .001) in detecting nCoV-19 infection because of the low efficiency of viral RT-PCR test.

Consequently, in screening of surgical patients, chest CT could be useful in decreasing the number of false-negative swab patients and to early individuate patients with uncertain COVID-19 infection who need to be isolated from negative patients.

On the other side, Bernheim et al. [20] analyzed data about 121 symptomatic COVID 19 patients and showed that 20/36 (56%) of patients had a normal CT in the early phase of the infection. CT pathological findings were more frequent when CT was performed later during the disease, including consolidations, bilateral and peripheral disease, greater total lung involvement, linear opacities, “crazy-paving” pattern and the “reverse halo” sign. Bilateral lung involvement was observed in 10/36 (28%) “early” patients, 25/33 (76%) “intermediate” patients and 22/25 (88%) patients in overt advanced viral pneumonia.

In the lack of recommendations about the screening of surgical patients for COVID-19 infection admitted in the ED with acute abdomen, many international societies of surgeons suggested to perform a swab test at admission and to complete the abdomen CT scan with a chest scanning for all patients.

By the time, it could not be viable because of the increasing number of symptomatic patients needing for a chest CT to assess the severity of the COVID-19 pneumonia and the overwhelmed ED. Furthermore isolation and barrier procedures were necessary in the radiology department to protect both the high exposed staff and other patients in the hospital. Consequently, radiology departments were re-organized in clean and contaminated areas and infection control measures had been implemented including providing adequate standard protective equipment, training staff, and instituting proper emergency plan to prevent intradepartmental spreading of infection such as waiting a safe period for air exchange and decontamination of the room and surfaces in the radiology department, increasing timing for a radiological examination [21].

Even if an abdominal CT scan is always performed in acute abdomen with clinical and biological signs of gravity, adding a chest CT could require more occupational time of the radiological room, a different acquisition protocol of images for the radiologist and an environmental risk of dissemination of the virus due to the inspiration and expiration requested to the patient during examination.

Furthermore, according to available evidence, CT scan used as a screening tool has some shortcomings, such as the overlap of imaging findings with other respiratory diseases and at very early stage of infection, the absence of any abnormalities [13, 15, 20]. Therefore, CT is not helpful as a screening tool, except when combined with other epidemiological, clinical, laboratory and RT-PCR test information to confirm the diagnosis and assess the severity of the pneumonia, even if CT scan results are available in a shorter waiting time than swab tests.

Other radiological imaging techniques, such as chest X-ray (CXR) and lung US, may be advocated in the screening of surgical patients, both to decrease the number of chest TC and in case of contraindication or unavailability of CT, to confirm the COVID-19 pneumonia.

Common CXR findings are bilateral interstitial pattern/ground-glass opacities, with isolated focal infiltrate making the diagnosis less likely.

In clinical practice, the CXR may be normal early in the disease course, so a normal x-ray does not rule out the diagnosis, exactly like a chest CT scan.

With the purpose to describe the time course and severity of the CXR findings of COVID-19 pneumonia and correlate radiological findings with RT-PCR testing for SARS-Cov-2 nucleic acid, a retrospective study was carried out in a cohort of 64 COVID-19 patients and the CXR demonstrated lower sensitivity of 69% [95% CI: 56–80%], compared with 91% [95% CI: 81–96%] (*p* = 0.009) for initial RT-qPCR. In this study, CXR abnormalities preceded positive RT-PCR in only 6/64 (9%) patients; moreover, common CXR findings mirror those described for CT that are bilateral, peripheral, consolidation and/or ground-glass opacities. The severity of CXR findings peaked at 10–12 days from the date of symptom onset [22].

The lung point-of-care ultrasound (POCUS) is reported to be useful in confirming clinical suspicion of COVID 19 infection in patients who showed negative RT-qPCR.

Vetrugno and Coll [23] reported that normal lung US shows A (complete or partial) lines that are a repetition of the pleural line at the same distance from the skin to the pleural line, indicative of air below the pleural line, corresponding to the parietal pleura, and B lines that arise from the pleural line and move in concert with a sliding lung and described as hyperechoic laser-like artefacts that resemble a “comet tail”. In pathological lungs, A lines are generally not present and B lines are associated with an interstitial syndrome and diminished lung aeration. B confluent lines appearing as a “white lung” (called also glass-rockets) are equivalent to CT ground-glass opacities that suggests a more severe loss of lung aeration. Lung consolidations are associated with hepatization of lung parenchyma with or without air bronchograms and suggest major loss of lung aeration.

Buonsenso et al. [24] were the first to clearly describe lung US signs suggestive for interstitial-alveolar damage showing on the anterior and posterior hemi-thorax bilaterally, an irregular pleural line with small subpleural consolidations, areas of white lung and thick, confluents and irregular vertical artefacts (B lines) and spared areas bilaterally, mixed with pathological areas.

The early US pulmonary manifestations of COVID-19 pneumonia are patchy distribution of interstitial artefactual signs (single and/or confluent vertical artefacts and small white lung regions) that will extend these patterns to multiple areas of the lung surface. The further evolution is represented by the appearance, still patchy, of small subpleural consolidations with associated areas of white lung. The evolution in consolidations, especially in a gravitational position, with or without air bronchograms, and their increasing extension along the lung surface indicate the evolution toward the phase of respiratory insufficiency that requires invasive ventilatory support [25].

Soldati et al. [25] reported that lung US has high sensitivity for detecting pleural thickening, sub pleural consolidation and ground-glass opacification equivalent in CT scan and suggested an acquisition protocol that would provide:

- The use of a convex or linear transducers. The latter are preferable to study the detail of the pleural and subpleural alterations.
- The use of a single–focal point modality (no multi-focusing), and set the focal point on the pleural line.
- Preferably, US scans need to be intercostal (not orthogonal to the ribs) to cover the widest surface possible with a single scan.

Ideally, 16 areas, if it is possible, should be evaluated: anterior mid-clavicular (apical, medial, and basal), right and left; posterior para-spinal (apical, medial, and basal), right and left; and lateral axillary (apical and basal), medial right and left, to study the extent of the lungs surface affected.

Advantages from the use of lung US as first radiological diagnostic investigation in suspected COVID-19 patients are [26–29]:

- US portability and bed-side evaluation that could decrease the virus exposure of healthcare personnel and environmental contamination derived from moving the patient to the radiology unit
- Easier sterilization of the device due to smaller surface areas
- Higher sensitivity (80%) than CXR (no more than 60%) to discriminate a bacterial pneumonia from a non-bacterial infection
- US radiation free
- US instrument costs

Lung POCUS limitations can be:

- The difficulty to detect a centrally located consolidation from bacterial superinfection;
- The inability to discern the chronicity of a lesion, limiting its power of early COVID19 diagnosis in the population with preexisting pulmonary conditions.

Given the limits of the currently used nucleic acid detection and CT scan for the diagnosis of COVID-19 used as screening tools, point-of-care test (POCT) of IgM/IgG and ELISA kits for SARS-CoV-2 have been developed to help in the detection of infected patients in the emergency setting.

In accordance with other acute viral infections, the antibody profile against SARS-CoV-2 has a typical pattern of IgM and IgG production. The SARS-specific IgM antibodies disappear at the end of week 12, while the IgG antibody can last for a long time [30–33].

Li et al. [30] studied the host humoral response against SARS-CoV-2 including IgA, IgM and IgG response by using an ELISA-based assay on the recombinant viral nucleocapsid protein and reported that the median duration of IgM and IgA antibody detection were 5 days (IQR 3–6), while IgG was detected on 14 days (IQR 10–18) after symptom onset, with a positive rate of 85.4%, 92.7% and 77.9%, respectively. The positive rates of IgM antibodies were 75.6% in confirmed cases and 93.1% in probable cases. Authors demonstrated also that the detection efficiency by IgM ELISA is higher than that of RT-PCR method after 5.5 days of symptom onset. The positive detection rate is significantly increased (98.6%) when IgM ELISA assay was combined with PCR for each patient compared with a single RT-PCR test (51.9%).

Moreover, the IgM-IgG combined assay was demonstrated to have better utility and sensitivity compared with a single IgM or IgG test.

On the other hand, controversial data are reported about the use of IgM/IgG rapid test in the triage of patients admitted to the ED.

Li et al. [32] reported an overall testing sensitivity and specificity of 88.66% and 90.63%, respectively.

Focusing on acute patients, Cassaniti et al. [34] reported a sensitivity and specificity of 18.4% and 91.7%, respectively, of the IgM/IgG rapid test while NPV was 26.2%, and PPV was 87.5%, revealing a very poor sensitivity (less than 20%). Indeed, the majority of patients that tested positive for COVID-19 by RT-qPCR would have been identified as negative using only the rapid serological assay, leading to a misdiagnosis of COVID-19 disease in the majority of patients.

### Is it necessary to delay the surgical procedure for a suspected COVID-19 patient until a RT-PCR swab test result is available?

#### Statement 2.1

All acute surgical patients should complete preoperative COVID-19 screening that includes RT-PCR nasopharyngeal swab test and chest CT scan, when it is available, or a CXR, or lungs US in the ED, whether they are symptomatic or not, to control the in-hospital spreading of SARS-CoV-2 (QoE moderate B).

#### Statement 2.2

Chest imaging such as a baseline CT scan, a CXR or a lungs US, depending on the availability, are useful diagnostic tools in the unavailability of RT-PCR swab test result to detect potentially infected patients (QoE moderate B).

#### Statement 2.3

If chest radiological evaluation by CXR, or chest CT scan or lungs US, is inconclusive and the patient needs for immediate surgery, he has to be treated as a COVID-19 patient to limit the risk of contagion and the spreading of the SARS-CoV-2 in the operating theatres (QoE moderate B).

#### Statement 2.4

After surgery, the uncertain patient has to be isolated as long as the RT-PCR test result is obtained, to be admitted in a COVID (+) or (−) ward. If it is positive, it is recommended repeating the swab test for confirmation. In patients with confirmed COVID-19 diagnosis, the laboratory evaluation should be repeated to evaluate for viral clearance prior to being released from isolation (QoE moderate B).

#### Statement 2.5

Timing of Acute Care Surgery (TACS) classification system could be a valid tool to evaluate timing of surgery and severity of the surgical disease (QoE low C).

#### Recommendations 2

We recommend completing the COVID-19 screening (RT-PCR nasopharyngeal swab test + chest imaging) for all acute surgical patients before admission in the surgical ward or operating theatre. If the RT-PCR swab test result is not available to confirm the diagnosis, the patient needs to be isolated and treated such as a COVID-19 (+) patient with all the mandatory precautions. The acute care surgeon is the only responsible for the decision of delaying a surgical procedure in the emergency setting during the pandemic. The TACS classification is a good tool to evaluate timing of surgery. According to this classification, surgery cannot be postponed for class 1 (immediate surgery) and class 2 (surgery in 1 h, as soon as possible) patients even if diagnosis of COVID-19 is not yet confirmed by the RT-PCR swab test (strong recommendation based on moderate-level evidence 1B).

#### Summary of evidence and discussion

The RT-PCR in respiratory tract samples is the current gold standard method for the diagnosis of COVID-19 infection but it could be time consuming because of the number of test requested and the availability of specialized operators and machines, when the rapid diagnosis is needed for fast intervention decisions. Generally, in very urgent cases, RT-PCR test can be obtained in 4–6 h; in other cases, test can be obtained in 48 h.

This is according to the availability and contraindications, if they are present, to performing radiological imaging, chest CT scan, or lung US, if emergency physician or surgeon is skilled to perform it, or CXR can help in confirming the diagnosis of COVID-19 pneumonia in surgical patients requiring immediate surgical procedure, showing pathognomonic features.

During this pandemic, the decision of operating or delaying the surgical procedure depends on the emergency surgeon’s evaluation.

If an urgent surgical procedure is necessary (life-threatening complication, high-risk patients, haemodynamic compromise, or shock), the emergency surgeon should check promptly the availability of a dedicated operating room with functional and suitable human and technical resources for COVID-19 and negative patients.

Several authors [35, 36] proposed a risk-stratification method to evaluate the priority of a surgical procedure in this pandemic but not specifically focusing on emergency conditions.

Many international societies of surgeons recommended rescheduling non-urgent surgeries but e*lective non-urgent surgery* does not always mean optional surgery. Delaying some surgical procedures could become very harmful because the progression of the underlying abdominal disease that can lead to life-threatening complications.

Focusing on emergency setting, early clinical diagnosis, adequate source control to stop ongoing contamination, appropriate antimicrobial therapy and prompt resuscitation in critically ill patients are the cornerstones in the management of intra-abdominal infections. Timing of surgical intervention is critical for outcomes of patients diagnosed with surgical emergencies. Under this period of limited access to hospital resources, the triage of the patients is fundamental to assess the severity of the intra-abdominal disease underlying the acute abdominal pain. Emergency surgeons have to decide for a non operative (NOM) or operative management of a surgical disease, such as it is recommended in international guidelines.

The haemodynamic stability or instability after adequate resuscitative maneuvers remains the main tool to risk-stratify patients for immediate surgery or not.

Furthermore, general, or more specific, clinical scores (such as for example the American Society of Anaesthesiologists’ (ASA) score, Alvarado’s score in case of acute appendicitis, SOFA for sepsis), the age of the patient and the presence of comorbidities, such as obesity, diabetes and COPD, can assist the emergency surgeon’s decision making process, associated with clinical (signs of localized or generalized peritonitis at abdominal examination) and biological (inflammatory biomarkers such as C-reactive protein, procalcitonin, lactates) parameters.

The WSES [37] proposed the Timing of Acute Care Surgery (TACS) classification to prioritize patients admitted in the ED with a potentially surgical condition. TACS is a color-coded triage system of acute care surgery cases based on simple haemodynamic and clinical data, such as it is shown in Table 2, to assist in evaluating patients when multiple patients require emergency surgery or limited resources are available.

In this COVID-19 pandemic, these criteria could guide the acute surgical teams to properly tag each patient to the timing of surgery.

**Table 2:**
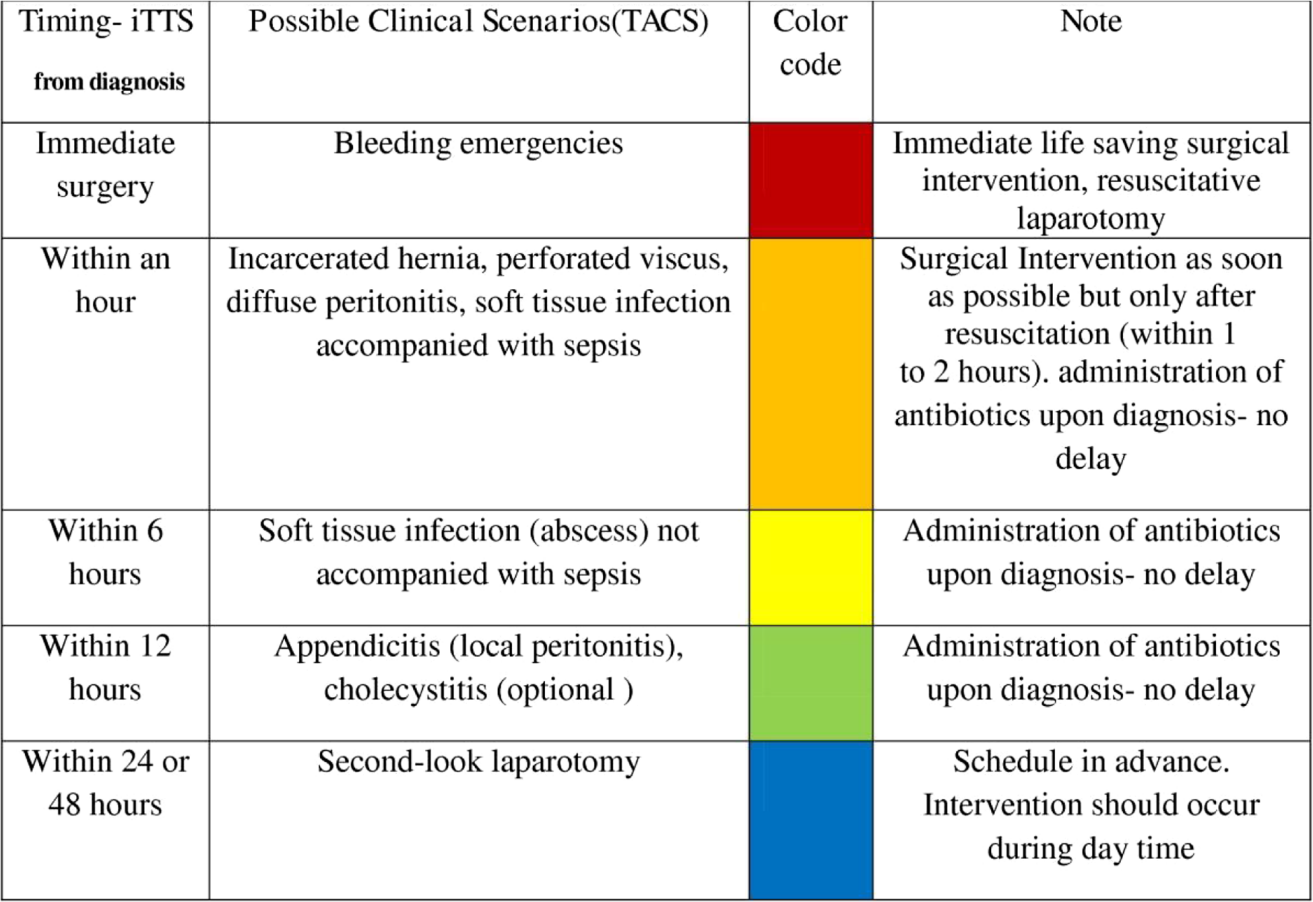
Timing of Acute Care Surgery (TACS) classification

Since now, the TACS classification is the only available tool to risk-stratify patients presenting with an emergency disease.

The validity of the TACS classification system was evaluated in a tertiary public hospital of a developing country, and data showed that TACS rating improves time for surgeries classified as yellow (ideal time to surgery within 6 h) [38].

Some authors decided to develop a more functional and robust system six-level classification, the Non-Elective Surgery Triage (NEST), allowing prioritization based on physiologic state and disease process, but no data are available about its application [39].

### In case of RT-PCR test unavailability and negative chest CT scan, suspected COVID-19 patients have to be operated using the operating theatres’ procedures procedures for overt COVID-19 patients?

#### Statement 3.1

RT-PCR test remains the reference standard to make a definitive diagnosis of COVID-19 infection and to manage the patient and resources in the correct way (QoE high A).

#### Statement 3.2

The emergency physician may identify high-risk COVID-19 patients investigating the presence of typical clinical symptoms, laboratory test results and/or epidemiological risk factors as suggested by the WHO, but RT-PCR test confirmation is mandatory to make diagnosis of viral infection (QoE moderate B).

#### Statement 3.3

Negative chest CT scan is not sufficient to exclude the diagnosis of COVID-19 infection, above all in the early phase of the infection (QoE low C).

#### Statement 3.4

In case of unavailability of the RT-PCR test, the surgical patient has to be considered potentially infected and managed like a COVID-19 (+) patient (QoE moderate B).

#### Recommendations 3

If it is not possible to confirm diagnosis of COVID-19 disease in an acute surgical patient by RT-PCR swab test, we recommend managing the patient such as he/she is COVID-19 (+) with all the mandatory precautions against viral infection, which include all the protective measures and a dedicated pathway for the operating room, to decrease the risk of environmental contamination and health personnel exposure. If a dedicated pathway for COVID-19 (+) patients is not available in the hospital, it should be an option to transfer haemodynamic stable suspected patient to the nearest COVID-19 HUB hospital for the appropriate management (strong recommendation based on the moderate level of evidence 1B).

#### Summary of evidence and discussion

Actual diagnosis of COVID-19 infection is made through:

- *COVID-19 RT-PCR test* that provides nucleic acid detection in the nasal and throat swab sampling, indicated in early stage of the infection, or other respiratory tract samplings
- *Chest imaging* that includes chest radiograph, CT scan or lung ultrasound demonstrating bilateral opacities (lung infiltrates > 50%), lobar or lung collapse. Multiple patchy ground-glass opacities in bilateral multiple lobular with periphery distribution are typical chest CT imaging features of COVID-19 pneumonia.

Several authors reported that a patient with RT-PCR-confirmed COVID-19 infection may have a normal chest CT at admission.

Fang et al. [19] reported one of 51 (2%) patient imaged 3 days ± 3 after symptom onset with normal CT.

Bernheim et al. [20] showed that 20 (56%) of 36 patients imaged 0–2 days after symptom onset had normal CT.

Ai et al. reported [40] 21 of 601 (3%) RT-PCR-positive patients with clinical symptoms had normal CT scans.

In contrast, Pan et al. [41] reported 4/21 (19%) patients with first normal CT had lung abnormalities on the follow-up CT approximately 4 days later.

These data confirm that a normal chest CT scan cannot exclude the diagnosis of COVID-19, especially for patients with early onset of symptoms or asymptomatic [13, 15, 19, 20].

Therefore, RT-PCR test remains the reference standard to make a definitive diagnosis of COVID-19 infection despite the false-negative rate.

Anyway, emergency physicians may identify high-risk COVID-19 patients by investigating the presence of typical symptoms and/or epidemiological risk factors as suggested by the WHO, but RT-PCR confirmation is mandatory to make diagnosis of viral infection and to manage the patient and resources in the correct way [3].

In case of unavailability of the RT-PCR test, the surgical patient has to be considered potentially infected and to be treated exactly as a COVID-19 patient, requiring all the protective measures to decrease the risk of environmental contamination and health personnel exposure and a dedicated pathway for the operating room.

Negative baseline chest CT scan is not sufficient to exclude the diagnosis of COVID-19 infection.

If it is not possible to diagnosis COVID-19 infection in a stable potentially surgical patient, it would be better to consider transferring the patient to the nearest COVID-19 HUB hospital for the management.

### In case of RT-PCR swab test unavailability and chest CT scan unavailability, suspected COVID-19 surgical patients have to be operated using operating room procedures for overt COVID-19 patients?

#### Statement 4.1

Diagnosis of COVID 19 disease is confirmed through the RT-PCR test (QoE moderate B)

#### Statement 4.2

Each surgical patient might be considered suspected for COVID-19 disease if clinical signs, imaging features at CXR or/and lungs US or/and chest CT scan and laboratory tests results are compatible with a SARS-CoV-2 infection (QoE moderate B).

#### Statement 4.3

The COVID-19 screening includes RT-PCR swab test and a chest radiological imaging that could be CXR or lungs US in the unavailability of CT scan (QoE moderate B).

#### Statement 4.4

If a surgical patient cannot complete the screening for COVID-19 disease and requires immediate surgical procedure, he/she should be managed with all the mandatory precautions against COVID-19 infection (QoE low C).

#### Statement 4.5

If the RT-PCR swab test is positive, the surgical patient has to be managed as a COVID-19 patient. The chest imaging is useful to assess the severity of the pneumonia (QoE high A).

#### Recommendations 4

In case of RT-PCR test and chest CT scan unavailability, we suggest screening the patient for COVID-19 pneumonia with CXR or lungs US that can help assessing viral pneumonia radiological signs and severity, exactly such as a chest CT scan, before surgery. In this case, SARS-CoV-2 infection can only be suspected (weak recommendation based on the low level of evidence 2C).

We recommend confirming COVID-19 disease with the RT-PCR swab test as soon as possible; if it is not possible, the suspected patient has to be managed as a SARS-CoV-2 infected one (strong recommendation based on the moderate level of evidence 1B).

#### Summary of evidence and discussion

Currently, in the unavailability of RT-PCR test and chest CT scan, we cannot identify COVID-19 patients but only put the suspicion for the infection in the presence of typical symptoms, including fever, dry cough, dyspnoea, myalgia, fatigue or hypolymphaemia, or epidemiological risk factors such as residence in or travel to an area with widespread community transmission or known contact [6, 7]. Therefore, surgical patients that cannot be screened have to be considered all potentially infected patients.

In case of unavailability of chest CT scan, we can complete COVID-19 screening with a CXR or lungs US.

Common CXR findings mirror those described for chest CT scan that are bilateral interstitial pattern/ground-glass opacities, with isolated focal infiltrate.

The CXR may be normal early in the disease course but a normal CXR does not rule out the diagnosis, exactly like a chest CT scan. CXR abnormal findings peaked at 10–12 days from the date of symptom onset [22].

In case of positive RT-PCR test and negative CXR, the patient has to be managed as a COVID-19 (+).

Chest radiological imaging in COVID-19 (+) patients helps the assessment of the severity of the pneumonia and the necessity to admit the patient in ICU.

If a surgical procedure is mandatory, the emergency surgeon should check the availability of a dedicated OR with functional and suitable human and technical resources and of the PPE necessary for protecting both patients and staff from intra-hospital transmission of the virus.

### Are emergency surgery indications for a confirmed COVID-19 patient different?

#### Statement 5.1

Indications for a surgical procedure are not different in confirmed COVID-19 patients (QoE moderate B).

#### Statement 5.2

Current data about outcome of surgery in COVID-19 patients showed a higher morbidity and mortality rate in comparison with negative patients (QoE moderate B).

#### Statement 5.3

The risk of environmental contamination and virus exposure in operating room related to the surgical management of a confirmed COVID-19 patient is high in the lack of trained health staff and personal protective equipment (QoE moderate B).

#### Statement 5.4

During COVID-19 pandemic, it is fundamental to carefully evaluate case by case the necessity for immediate surgical or non operative strategies, as recommended in international guidelines (QoE moderate B).

#### Recommendations 5

In evaluating the necessity to perform emergency surgery in COVID-19 (+), we recommend complying with international guidelines about immediate surgery or non operative strategies, evaluating case by case and resources. According to TACS classification, class 1 and 2 patients require surgical treatment in a very short delay (strong recommendation based on the moderate level of evidence 1B).

#### Summary of evidence and discussion

Indications for an urgent surgical procedures are not different in confirmed COVID-19 patients; the difference lies in the access to the resources (restricted access to the operating theatres, limited number of beds in ICUs, respirators and transfusion capacity) under the peak of this pandemic and the risk of virus dissemination in the environment and in OR, in the lack of dedicated pathway and staff.

The main objective of the ES is not to delay surgery to decrease morbidity and mortality rates in patients in whom a surgical procedure is mandatory.

Consequently, triage of the infected patients with TACS classification remains the cornerstone of the management of intra-abdominal surgical diseases. The assessment of the severity of the surgical disease and of the viral pneumonia enables ES to decide on the management of the patient, complying with international guidelines.

On the assessment of the severity of the COVID-19 pneumonia, patients can be classified in [11]:

- Patients with *mild illness*: this group of patients does not need ventilatory support or admission in ICU.
- Patients presenting with *moderate viral pneumonia*: they could require non-invasive ventilatory support.
- Patients with *severe pneumonia*: this is a group of critically ill patients that could present with Acute Respiratory Distress Syndrome (ARDS) and need to be admitted in the ICU to receive ventilatory support or extracorporeal membrane oxygenation (ECMO).

In the initial evaluation of a patient presenting with acute abdomen, the haemodynamic status remains the main tool to risk-stratify patients in need of immediate surgery.

Then, if the intra-abdominal infection is uncomplicated (i.e. involving the organ and not the peritoneum), NOM could be a valid option, to be considered on a case-by-case basis, according to WSES guidelines [42].

In case of NOM, it is crucial to plan close clinical and radiological surveillance at 12–24-h intervals from the beginning of the intravenous antibiotic therapy until the situation is under control. If the patient presents with persistent abdominal pain, fever or signs of shock, surgical treatment cannot be postponed.

Let us critically review the management of the most common surgical diseases, on the basis of the currently evidence.

In case of diagnosis of *acute appendicitis*, the “gold standard” treatment is laparoscopic appendectomy in all patients. In this specific scenario, caution might be taken in the choice of the laparoscopic approach because of concerns about the presence of the virus in the pneumoperitoneum, if it is not possible to decrease the risk of dissemination of the virus in OR. However, NOM with antibiotic therapy has proven to be successful in uncomplicated appendicitis. The Jerusalem WSES guidelines and its 2020 update [43] considered appropriate the choice of NOM in selected patients (both adult and children) to treat uncomplicated acute appendicitis. Patients and surgeons must be aware of a risk of recurrence of up to 39% after 5 years. Most recent data from meta-analyses of RCTs showed that NOM with antibiotics achieves a significantly lower overall complication rate at 5 years and shorter sick leave compared to surgery. Anyway, in this scenario, the risk of recurrence does not appear to be pivotal in the decision making and an “antibiotic first” policy can be a safe tool to avoid surgery for uncomplicated acute appendicitis during the pandemics, and postpone it, eventually, to treat a recurrence.

Similarly, under COVID-19 pandemic, patients presenting complicated appendicitis with a well-defined abscess in the right iliac fossa can be managed with percutaneous drainage, if it is available, associated with IV antibiotics. Patients with evidence of perforation may be managed with percutaneous drainage or operation based on patient condition, or if unfit for surgery. Patients who fail NOM should proceed to surgery expeditiously [43].

In case of diagnosis of a*cute cholecystitis*, the laparoscopic cholecystectomy remains the treatment of choice [44]. In particular, early laparoscopic cholecystectomy (LC) is better than delayed LC. Multiple RCTs have shown early cholecystectomy to be associated to a shorter hospital stay without any significant difference in the complication rate or conversion, when compared with delayed cholecystectomy. However, the recommendation of early surgery could be challenged due to the pandemic-related restrictions. Managing the infection with IV antibiotics and analgesics in order to delay surgery should be seriously considered in such a context. Percutaneous cholecystostomy (PC) with IV antibiotics has been advocated as an alternative to surgery in critically ill patients. PC involves the sterile placement of a tube into the gallbladder for external drainage of gallbladder contents. The procedure can be performed using US or CT scan guidance. US is the modality of choice because of the readily availability of the device, the portability of the machine, and real-time imaging during the procedure that can be performed safely at the bed side of the patient under adequate sedation and local anaesthesia [45].

The objectives of a PC are to decompress the gallbladder through a drainage left in place and to obtain cultures from the bile for appropriate antibiotic therapy.

Actually, the 2016 WSES guidelines did not recommend it as an alternative to LC except in the most unfit patients due to the significantly higher mortality rate. The recent CHOCHOLATE trial confirmed that finding and was interrupted due to the poor results in the PC group [46].

In case of diagnosis of (jejunal or colonic) acute diverticulitis (AD) [47], the treatment of choice for uncomplicated AD is NOM with IV antibiotics, considering transition to oral antibiotics as soon as possible. Patients who present with generalized peritonitis should undergo urgent surgical treatment. In case of jejuno-ileal diverticulitis, there are no consensus and no recommendations; anyway, surgery is the preferred treatment because of high mortality and morbidity related to the severity of the disease [48].

Patients presenting with Hinchey class I and II diverticulitis should be managed with percutaneous drainage in addition to antimicrobial therapy if a large (> 4 cm) abscess is visualized on CT scan. If percutaneous drainage is not available, the patient could be managed with antibiotics but surgery should be considered if there are signs of sepsis or shock. Patients who fail NOM should be expedited for surgery. Patients with pneumoperitoneum (free air distant to the sigmoid) and peritonitis should be considered for surgery. In this scenario, the surgical options are:

- Hartmann’s procedure (HP) for managing diffuse peritonitis in critically ill patients and in selected patients with multiple comorbidities;
- Primary resection with anastomosis with or without a diverting stoma in clinically stable patients with no major comorbidities.

Emergency laparoscopic sigmoidectomy should be avoided, especially if very long operative duration is expected.

In the presence of uncomplicated jejunal diverticulitis, NOM with IV antibiotics could be an effective therapeutic option, if patient does not present signs of haemodynamic compromise.

If jejunal diverticulitis is complicated by an abscess, the radiological drainage of the intra-abdominal collection could be a valid choice and at the same time the cultural analysis of the liquid can give useful information to adapt antimicrobial treatment.

Patients who fail NOM have to be operated without delaying surgery. Intestinal resection with anastomosis is the best surgical option in stable patients. If patients present with signs of shock, haemodynamic instability and generalized faecal peritonitis, to avoid poor outcomes, intestinal resection with stoma creation is an option to take in consideration to minimize the occupational time of the OR.

In case of diagnosis of *colon obstruction or perforation* [49], left colonic obstruction would best be treated by a loop colostomy (short operative time) or HP. The latter should be preferred over simple colostomy to avoid longer hospital stay and multiple operations. On the other hand, the loop colostomy can temporarily treat the occlusion (bridge to surgery option), and definitive surgery can be planned in accordance with the management of the hospital resources under the COVID-19 pandemic.

Generally, WSES recommends reserving the loop colostomy for patients with unresectable tumors or too unfit for major surgery or even general anaesthesia. Colonic stent placement could have a role, but endoscopy can promote the spreading of the virus; consequently, WSES suggests to consider it in COVID 19 patients only if unfit for surgery and general anaesthesia [49].

Colonic resection and primary anastomosis, with or without loop ileostomy, should be the preferred option for uncomplicated malignant left-sided large bowel obstruction. However, it may increase operative time, with a high risk of viral exposure. Patients with high surgical risk would be better managed with HP.

In the absence of major caecal distension, bowel ischaemia or synchronous patent right colonic cancer, total colectomy should not be undertaken. In the case of obstructive right-sided colon cancer, right colectomy with primary anastomosis is the preferred option. An end ileostomy with colonic fistula represents a valid alternative when a primary anastomosis is deemed too hazardous.

For unresectable right-sided colon cancer, a side-to-side anastomosis ileotransverse internal bypass or a loop ileostomy could be performed.

In case of *adhesive small bowel obstruction (ASBO)* [50], even during the COVID-19 pandemic, NOM (i.e. nil by mouth, nasogastric decompression) should always be the first approach in patients unless there are signs of peritonitis, strangulation or bowel ischaemia. NOM is effective in approximately 70–90% of patients with ASBO. While evidence for the optimal duration of NOM is lacking, most authors consider up to 72 h as safe and appropriate.

In case of intestinal occlusion in incarcerated abdominal hernia [51], patients should undergo emergency hernia repair immediately when intestinal strangulation is suspected in order to avoid intestinal ischaemia. In case of an incarcerated inguinal hernia, local anaesthesia can be used (in the absence of bowel gangrene) to decrease the risk of aerosol spreading of the virus in the operating theatre.

In patients presenting with *perforated peptic ulcer (PPU)* [52], NOM could be considered in extremely selected cases where perforation has sealed as confirmed on water-soluble contrast study; the endoscopic treatment by clipping, fibrin glue sealing or stenting under this pandemic is to avoid either the high risk of failure or the risk of environmental contamination and healthcare personnel contagion.

In patients with significant pneumoperitoneum or extraluminal contrast extravasation or signs of peritonitis, immediate surgery is mandatory and the laparoscopic approach is usually the first choice. During COVID-19 pandemic, if appropriate laparoscopic skills to minimize the occupational time of the OR and general anaesthesia, and in the absence of the equipment to perform a safe laparoscopy, the open approach is recommended, above all in unstable patient.

In patients with *bleeding peptic ulcer (BPU)* [52], WSES recommends endoscopic treatment as first line approach to achieve hemostasis and reduce re-bleeding, the need for surgery, and mortality. The trans-catheter angioembolization could be a valid alternative in this pandemic where resources are available, but in unstable patients with refractory BPU, surgery should be mandatory, by laparoscopic approach if there is the expertise and the necessary equipment or in open approach.

### Are emergency surgical procedures for confirmed COVID-19 patients different?

#### Statement 6.1

SARS-CoV-2 is presumed to spread primarily via respiratory droplets and aerosols and close contact, but the virus can be isolated also in the faeces and biological fluids of the infected patient (QoE high A).

#### Statement 6.2

Human coronaviruses can persist on inanimate surfaces such as metal, glass or plastic for up to 9 days (QoE high A).

#### Statement 6.3

Aerosol-generating procedures (AGP) are considered responsible for the dissemination of the SARS-CoV-2 virus in the hospital (QoE moderate B).

#### Statement 6.4

Performing or being exposed to a tracheal intubation without adequate PPE is the main risk factor for healthcare workers SARS-CoV-2 infection (QoE high A).

#### Statement 6.5

Laparoscopic approach has been advocated such as a high-risk AGP because of the artificial pneumoperitoneum and smoke generated from the surgical devices (QoE very low D).

#### Statement 6.6

Laparotomy such as laparoscopy should be considered a high-risk procedure that can be implicated in the intra-hospital dissemination of the virus because of the higher exposure to biological fluids, surgical smoke generated with the use of electrocautery (QoE low C).

#### Statement 6.7

The laparoscopic approach could have the advantage of decreasing the length of hospital stay of an asymptomatic COVID-19 patient and the risk of in-hospital infection of a negative patient, in a period of limited availability of beds (QoE very low D).

#### Statement 6.8

Contraindications to laparoscopy are not evidence-based since aerosolization is produced during both open and laparoscopic surgical procedures. However, personal protective equipment is the key for prevention (QoE high A).

#### Statement 6.9

The emergency surgeon has the responsibility to evaluate if a safe surgical procedure is possible considering the restricted access to resources and the safety of surgical staff and of patient (QoE high A).

#### Recommendations 6

If an immediate surgical procedure needs to be performed, whether laparoscopic or via open approach, we recommend doing every effort to protect the operating room staff, for the safety of the patient (strong recommendation based on low-level evidence 1C).

To perform a safe surgical procedure, we recommend having a trained staff, wearing the necessary PPEs and an established protocol for the preoperative, perioperative and postoperative management of the COVID-19 surgical patient (strong recommendation based on low-level evidence 1C).

We recommend to not be present during the intubation and extubation maneuvers, if it is possible (strong recommendation based on moderate-level evidence 1B).

We recommend being careful in the establishment and management of the artificial pneumoperitoneum and in the management of the hemostasis and of incisions to prevent any loss of biological fluids and contamination of the surgical staff (strong recommendation based on low-level evidence 1C).

We recommend using of all available devices to remove smoke and aerosol during the surgical procedure in both laparoscopy and open approaches (strong recommendation based on low-level evidence 1C).

If it is not possible to perform surgery in a safe and protected environment, we recommend not underestimating the highest risk of contamination and infection for healthcare workers and dissemination of the virus in the hospital and to consider transferring haemodynamically stable patients in a COVID HUB hospital for the appropriate management (strong recommendation based on low-level evidence 1C).

#### Summary of evidence and discussion

Patients who have failed NOM for a surgical condition or who present with haemodynamic instability should be considered for immediate surgery.

The surgical procedure has to be organized according to the protocol for infection in-hospital control.

In evaluating the appropriate surgical technique, concerns have been expressed about the use of the laparoscopic approach.

According to available data, SARS-CoV-2 is presumed to spread primarily via respiratory droplets and aerosols and close contact. Human coronaviruses, such as SARS-CoV and MERS coronavirus, or endemic human coronaviruses can persist on inanimate surfaces such as metal, glass or plastic for up to 9 days [53, 54].

In a brief report, Wang and Du [55] defined aerosols such as particles formed by solid or liquid particles dispersed and suspended in the air. These may contain soil particles, industrial dust particles, particulates emitted by automobiles, bacteria, microorganisms, plant spore powders or other components. When a person infected with the virus coughs, sneezes, breathes vigorously or speaks loudly, the virus will be excreted from the body and may dissolve with the aerosols, becoming bioaerosols. The particles in a bioaerosol are generally 0.3–100 μm in diameter but only the respirable size fraction of 1–10 μm is of particular concern. Bioaerosols ranging in size from 1.0 to 5.0 μm usually remain in the air whereas larger particles are deposited on surfaces. Droplets of saliva are discharged by people sneezing or coughing and their particle size is generally 1–5 mm. These spread in a space of about 1–2 m from the source of the infection. However, the aerosol can travel hundreds of meters or more. On the way of the SARS experience, researches have proved that aerosols are involved in the spread of SARS, MERS, H1N1 and, by extrapolation, COVID-19. As reported for SARS, SARS-CoV-2 seems to spread mostly by direct exposure to infectious droplets and secretions but further evidence has indicated that indirect transmission by environmental contamination could be responsible in cases of nosocomial transmission of the virus [54].

Van Doremalen et al. have demonstrated that SARS-CoV-2 is more stable in aerosols and on various surfaces (plastic and stainless steel) under experimental conditions than SARS-CoV-1 and that it can remain viable and infectious in aerosols for hours and on surfaces, for days (depending on the inoculum shed) [56].

Three mechanisms have been described for the production of smaller sized respiratory particles (aerosols) that, if inhaled, can deposit in the distal airways and include laryngeal activity such as talking and coughing, high velocity gas flow, and cyclical opening and closure of terminal airways. Sneezing and coughing are effective aerosol generators, but all forms of expiration produce particles across a range of sizes. The 5-μm diameter threshold is used to differentiate droplet from airborne [56].

Potential aerosol-generating procedures (AGP), as defined by the WHO [57] include endotracheal intubation and related procedures (e.g. manual ventilation, suctioning), cardiopulmonary resuscitation, bronchoscopy, surgery and autopsy.

AGP are considered responsible of the SARS-CoV-2 transmission among healthcare workers (HCW).

Tran et al. [58] carried out a systematic review to determine the clinical evidence for the risk of transmission of acute respiratory infections to HCW during AGP compared with the risk of transmission to HCW caring for patients not undergoing AGP and reported that some procedures potentially capable of generating aerosols have been associated with an increased risk of SARS transmission from SARS-CoV-infected patients to HCWs. Of the assessed procedures, including tracheal intubation, non-invasive ventilation, tracheostomy and manual ventilation before intubation, performing or being exposed to a tracheal intubation appeared to be the most AGP associated with SARS-CoV transmission.

For Wilson et al., there is no proven relation between any AGP with airborne viral content with the exception of bronchoscopy and suctioning and they suggest that several AGP may result in less SARS-CoV 2 aerosolization than a dyspnoeic and coughing patient [59].

During surgery, aerosolization can result from dissection with electrosurgical instruments, as the heat of such devices results in a plume of surgical smoke, in either open or laparoscopic approach.

Since COVID-19 outbreak, many authors suggested great care when carrying out a laparoscopic procedure in surgical patients with COVID-19, on the basis of a theoretical risk of occupational exposure and infection of the operating theatre staff, although to our knowledge, there has been still no study that has firmly confirmed the presence of the virus in theatre during laparoscopic procedures or in the artificial pneumoperitoneum [60–64].

Recently, the PCR test was used to confirm the presence of the SARS-CoV-2 in the peritoneal fluid in a case report [65].

Instead, there are studies that have demonstrated that electrosurgical devices can produce aerosolized bacteria and viruses including human immunodeficiency virus, human papillomavirus and hepatitis virus with a number of studies demonstrating a risk of oral papillomatosis due to occupational exposure during open surgery [64–71].

The aerosolization risk in laparoscopy is still unclear. A study by Giannella and colleagues reported traces of anaesthetic sevoflurane being also found, showing an unknown mechanism of passage between the lungs and the abdominal cavity [72]. By experience, we know that sevoflurane can be present in the air also during open procedures as well.

At early stage of this pandemic, in the lack of strong evidence, many international societies were nearly to prohibit laparoscopy, except in ultra-selected cases.

More carefully, several authors [60–64] proposed different techniques to perform a safe laparoscopy, based on the principle of limiting the leakage of gas. Recommended techniques include using constant pressure insufflators to reduce the aerosol effect of insufflation and central aspirator systems to drain the smoke. For example, some authors suggest using the closed circuit of pressurized intraperitoneal aerosol chemotherapy if available or connecting one of the laparoscopic ports to a water seal created with a sealed container using extension lines [60–64]. Before making the incision for retrieval of an operative specimen, the “gas” must be turned off and the pneumoperitoneum must be emptied by means of the negative pressure connected to the water seal [60–64]. Special attention should be paid to evacuating residual pneumoperitoneum from the container and the abdominal cavity before removing the trocars.

For some authors, during this crisis, a laparoscopic approach should be avoided because it could be associated with longer operative time (and therefore increased risk of exposure and occupational time of OR), especially in an emergency setting [63]. For others, laparoscopic procedures create a functional barrier between the surgeon and the disease because the abdomen is not opened, reducing exposure to the disease (including the dissemination of aerosol) compared with open surgery.

Considering available evidence since now, both laparoscopic and open approach could be considered AGP and could contribute to the environmental contamination and the virus exposure of the HCW.

Taking in consideration patient safety and infection prevention, on a case-by-case evaluation, the emergency surgeon has to select the appropriate surgical technique for that patient in that hospital.

The availability of the adapted surgical equipment, all PPE and trained HCWs are essential to perform a safe surgical procedure [69].

Generally, a number of strategies are employed in the hospital and in the operating rooms to minimize exposure to the virus and to decrease the risk of environmental contamination including negative-pressure ventilation (preventing cross-contamination between rooms), minimizing time and exposure during intubation, using surgical masks such as FFP2 (minimum) or FFP3, as well as smoke evacuation systems in case of a laparoscopic approach and a suction system to limit the exposure to surgical smoke while performing a laparotomy.

According to these data, we suggest, both in laparoscopic and in laparotomy, to carefully balance the risk of potential viral exposure and transmission in that particular situation and the clinical benefits of a minimally invasive approach or a laparotomy for that patient.

All mandatory precautions to perform a safe surgical procedure in a COVID-19 patient (confirmed or uncertain) are listed in the Table 3.

**Table 3:** Tips and tricks to perform a safe surgical procedure in COVID-19 era

Performing a safe laparoscopic approach	Performing a safe laparotomy
Check if a closed suction system is available	Avoid huge incision causing loss of biological fluids and staff contamination
Create suitable surgical incisions for the introduction of leak-free trocars such balloon trocars if available	Think to protect the incision with a double ring wound protector, if it is available in according to recommendations for SSI control
Be sure not to contribute in increasing the OR air contamination by creating a leak in the presence of smoke obstructing the intervention	The power settings of electrocautery should be as low as possible
Aspirate the entire pneumoperitoneum before making an auxiliary incision to extract the specimen, at the end of the procedure before removing the trocars or before converting the intervention to laparotomy	Avoid long dissecting times on the same spot by electrocautery or ultrasonic scalpels to reduce the surgical smoke
Keep intraoperative pneumoperitoneum pressure and CO2 ventilation at the lowest possible levels without compromising the surgical field exposure	Use the suction devices to remove the surgical smoke
Reduce the Trendelenburg position time as much as possible. This minimizes the effect of pneumoperitoneum on lung function and circulation, in an effort to reduce pathogen susceptibility	Special attention is warranted to avoid sharp injury or damage of protective equipment, in particular gloves and body protection
Avoid long dissecting times on the same spot by electrocautery or ultrasonic scalpels to reduce the surgical smoke	Minimize the use of drainage

Having reviewed the available literature on COVID-19, we concur that when considering the laparoscopic approach in an emergency, every effort should be made to limit the leakage of gas, but there seems no reason to abandon laparoscopic surgery over open surgery [72].

We recommend making modifications to surgical practice such as the use of smoke evacuation/suction and minimizing energy device/electrocautery use among other measures to minimize operative staff exposure to aerosolized particles.

Contraindications to laparoscopy are not evidence-based since aerosolization is produced during both open and laparoscopic surgical procedures. However, personal protective equipment is the key for prevention [72].

In the lack of PPE and general measures to prepare the operating theatre, such as summarized in the Table 4 for a COVID-19 patient, and in the impossibility to perform surgery in a safe and protected environment, we suggest not to perform surgery for the highest risk of virus exposure and environmental contamination and to consider to transfer the patient to an HUB COVID hospital.

**Table 4:** The checklist for the safe management of urgent surgical COVID-19 patients

Urgent surgical patients' management in COVID-19 era check list	Yes	No
Defined in-hospital route for patients with suspected or confirmed COVID-19		
Availability of all necessary PPE including FFP2 mask, eye protection, cap, oversized waterproof long-sleeved gown, knee-high shoe protection and gloves (always a double pair) and trained operating room staff		
Availability of a negative-pressure environment to reduce dissemination of the virus beyond the operating theatre or of a standard positive-pressure operating theatre with a high frequency of air renewal (25 times per hour) to reduce the viral load		
**In the operating theatre**		
The number of staff involved in any surgical procedure should be limited		
The name of all participating staff members should be recorded to facilitate contact tracing		
Theatre doors must be closed for the entire duration of the operation		
Movement of staff in and out of the operating theatre should also be restricted		
Only selected equipment and drugs should be brought into theatre to reduce the number of items that need to be cleaned or discarded following the procedure		
A runner, stationed outside the operating theatre, should be available if additional drugs or equipment are needed		
Anaesthetic monitors, laptop computers and ultrasonography machine surfaces should be covered with plastic wrap to decrease the risk of contamination and to facilitate cleaning		
The patient should be examined, induced and recovered in the operating theatre itself to restrict contamination to just one room		
The addition of an expiratory port with a bacterial/viral filter (e.g. HEPA filter) can reduce aerosol emission as well as the use of a closed tracheal suctioning system for aspiration of respiratory secretions		
The surgical team will don scrubs following the usual procedure for performing surgery but replacing the surgical mask with a FFP2 (minimum) or FFP3 mask, wearing high shoe protection and a waterproof gown. Eye protection (goggles) or facial protection (face mask) should be always worn		
**After the surgery**		
All staff have to shower and change into a clean set of scrubs before resuming their regular duties		
The PPE used must be disposed of inside the containers for special waste at risk of infection		
The name of all participating staff members is recorded to facilitate contact tracing		
The operating room must be sanitized as soon as possible *Human coronaviruses can be efficiently inactivated by surface disinfection procedures with 62–71% ethanol, 0.5% hydrogen peroxide or 0.1% sodium hypochlorite within one minute. Other biocidal agents such as 0.05–0.2% benzalkonium chloride or 0.02% chlorhexidine digluconate are less effective*		

A strategy for damage control surgery may have a role in haemodynamically compromised patients [73]. Patients treated with either damage control surgery or open abdomen will need to be admitted to the ICU. However, as ICUs are currently overwhelmed because of the pandemic, the indications for performing open abdomen surgery should be carefully evaluated on a case-by-case basis.

### Confirmed COVID-19 patients have a different low-molecular-weight heparin (LMWH) prophylaxis?

#### Statement 7.1

COVID-2019 infection can activate coagulation cascade through various mechanisms, leading to severe hypercoagulability. Early anticoagulation may block clotting formation and reduce microthrombus, thereby reducing the risk of major organ damages (QoE moderate B).

#### Statement 7.2

In confirmed COVID-19 patients, routine d-dimer testing on admission and serially during hospital stay should be considered to stratify the risk of venous thromboembolism (VTE). In the case of significantly elevated d-dimer levels (≥ 1.5–2.0 mg/L), pharmacological VTE prophylaxis should be initiated (QoE moderate B).

#### Statement 7.3

Prophylactic-dose LMWH should be initiated in all surgical patients with COVID-19 disease admitted to the hospital to decrease thromboembolic risk related to the infection and emergency surgery (QoE moderate B).

#### Statement 7.4

Prophylactic anticoagulation reduces the risk of VTE in acutely ill hospitalized medical patients when the risk of bleeding is acceptable (QoE moderate B).

#### Statement 7.5

Anticoagulant therapy mainly with LMWH appears to be associated with better prognosis in severe COVID-19 patients, according to the risk of surgical bleeding (QoE moderate B).

#### Statement 7.6

If pharmacological VTE prophylaxis is indicated, LMWH should be given at a dosage approved for high-risk situations. In case of contraindications for anticoagulation, physical measures should be used (e.g. medical compression stockings) (QoE moderate B).

#### Statement 7.7

Intensified VTE prophylaxis should be considered in patients with additional risk factors (e.g. body mass index > 30 kg/m^2^, history of VTE, known thrombophilia, active cancer) or requiring ICU admission or with rapidly increasing d-dimer levels, taking into account renal function and bleeding risk (QoE moderate B).

#### Statement 7.8

Following discharge from hospital, prolonged pharmacological VTE prophylaxis is reasonable in patients with persistent immobility, high inflammatory activity, and/or additional risk factors (QoE low C)

#### Statement 7.9

In hospitalized COVID-19 patients who develop VTE, especially in those requiring ICU admission, LMWH at therapeutic dosages may be considered the standard of care. In case of severe renal insufficiency, unfractionated heparin should be administered (QoE moderate B).

#### Recommendations 7

We recommend administering prophylactic anticoagulation with LMWH as soon as possible in COVID-19 surgical patients to reduce the thromboembolic risk related to the virus, sepsis and emergency surgery. The dosage of the anticoagulant therapy has to be adjusted according to the risk of surgical bleeding, renal function and weight of the patient (strong recommendation based on moderate-level evidence 1B).

If it is not possible to administer an anti-thrombotic prophylaxis, we recommend considering the intermittent pneumatic compression, in case of immobilized patient, and mobilizing the patient as soon as possible after surgery (strong recommendation based on the moderate level of evidence 1B).

#### Summary of evidence and discussion

The most consistent hemostatic abnormalities with COVID-19 patients include mild thrombocytopenia and increased d-dimer levels, which have been associated with a higher risk of requiring mechanical ventilation, ICU admission and death [74].

Available data suggest that COVID-19 disease may predispose to both venous and arterial thromboembolism due to excessive inflammation, hypoxia, immobilization and diffuse intravascular coagulation. In particular, critically ill patients with SARS-CoV-2 showed a high risk to present thromboembolic events.

Zhang et al. reported significant aberrant coagulation changes in critically ill COVID-19 patients admitted to the ICU: fibrinolytic degradation products (d-dimer and fibrinogen degradation products) were significant above normal range in almost all patients; the levels of fibrinogen and factor VIII coagulant activity were above normal range in the majority of patients; the natural anticoagulant activities were mildly lower. Nearly half of patients developed thrombotic events after admission to ICU. These findings implied that sustained hypercoagulable status and activation of coagulation system are hallmarks of critically ill COVID-19 and provided a strong evidence to support anticoagulation therapy in these patients [73].

Cui et al. [74] carried out an observational study in 81 COVID-19 patients admitted in the ICU and reported that the incidence of lower extremity deep venous thrombosis is 25% (20/81) and it may be related to poor prognosis. This group of patients showed a significant increase of d-dimer that could be considered a good index for identifying high-risk groups of venous thromboembolism (VTE) in COVID-19 patients.

Another observational study from the Netherlands examined the incidence of both venous and arterial thromboembolic events in 184 ICU SARS-CoV-2 patients. They observed 25 pulmonary embolism, and 3 ischemic strokes, and reported that VTE occurred in 27% of patients [95% CI: 17–37%] and arterial thrombotic events in 3.7% [95% CI: 0–8.2%]. The acute pulmonary embolism was the most frequent thrombotic complication (*n* = 25, 81%). Age and coagulopathy were identified such as independent predictors of thrombotic complications [75].

These rates are very high when compared to a recent RCT conducted on ICU (no COVID) patients that reported the incidence of VTE to be 9.4% [76]. It has been observed that in COVID-19 patients, during the development of dyspnoea and chest imaging changes from light to severe, the d-dimer increased from mild to significant, along with prolonged prothrombin time and gradual decrease of fibrinogen and platelet [75]. Recently, it has been reported that some of the non-survivors COVID-19 patients suffered from ischemic changes such as ecchymosis of the fingers and toes at the same time as the organ functions of the heart and kidneys became worse. Tang et al. [77] analyzed the coagulation parameters of 183 consecutive infected patients and investigated the differences between survivors and non-survivors. The overall mortality was 11.5%; the non-survivors revealed significantly higher d-dimer and fibrin degradation product (FDP) levels, longer prothrombin time and activated partial thromboplastin time compared to survivors on admission (*P* < .05); 71.4% of non-survivors and 0.6% survivors met the criteria of disseminated intravascular coagulation during their hospital stay.

It has been proven that the coagulation system can be activated by a variety of different viruses [78]. High plasma levels of pro-inflammatory cytokines (interleukin-2, interleukin-7, granulocyte colony-stimulating factor, IP10, MCP1, MIP1A and tumor necrosis factor-α) have been observed in critically ill COVID-19 patients admitted to intensive care units. This is consistent with a “cytokine storm” with the secondary development of a hemophagocytic lymphohistiocytosis [79].

While many pro-inflammatory cytokines trigger the coagulation system, Zhou and colleagues [80] showed that the increase in IL-6 was discrepant with the elevations in d-dimer; IL-6 levels appeared to increase only 13 days after disease onset, whereas d-dimer levels were already 10-fold increased by that time. This observation suggests that the very high d-dimer levels observed in COVID-19 patients are not only secondary to systemic inflammation, but also reflect true thrombotic disease, possibly induced by cellular activation that is triggered by the virus.

Whether the coagulation cascade is directly activated by the virus or whether this is the result of local or systemic inflammation is not completely understood, anyway, vascular endothelial damage in both small- and mid-sized pulmonary vessels was noted together with disseminated intravascular coagulation, deep vein thrombosis and pulmonary embolism resulting in pulmonary infarction in SARS-CoV and SARS-CoV-2 [81, 82]

Tang et al. [83] conducted a single-centre retrospective cohort study of 449 consecutive severe COVID-19 patients, defined as either a respiratory rate ≥ 30/min, arterial oxygen saturation ≤ 93% at rest or PaO2/FiO2 ≤ 300 mmHg and reported that prophylactic doses of heparins might be associated with improved survival (20%) in patients with evidence of sepsis induced coagulopathy (SIC). Of the 449 patients, 99 (22%) received heparin for 7 days or longer (low-molecular-weight heparin) LMWH in 94 patients, usually enoxaparin 40–60 mg/day, and unfractionated heparin in 5. Heparin was associated with lower 28-day mortality among the 97 patients with a SIC score ≥ 4, (40% vs 64%; OR, 0.37 [95% CI: 0.15–0.90]; *P* = 0.029), but not among the 352 patients with a SIC score, which includes prothrombin time, platelet count and sequential organ failure assessment (SOFA), < 4 (29% vs 23%; *P* = 0.42).

The WHO interim guidance statement recommends prophylactic daily LMWHs, or twice daily subcutaneous unfractionated heparin (UFH) [84].

If pharmacological prophylaxis is contraindicated, mechanical VTE prophylaxis (intermittent pneumatic compression) should be considered in immobilized patients [85]. Missed doses of pharmacologic VTE prophylaxis are common and are likely associated with worse outcomes.

The risk of VTE is increased in critically ill COVID-19 patients. Alterations in pharmacokinetics in this group of patients may necessitate anticoagulation dose adjustment, due to factors relating to absorption, metabolism and renal (or hepatic) elimination of these drugs in the setting of potential organ dysfunction.

The anti-thrombotic prophylaxis showed high rate of failure above all in COVID-19 patients admitted in ICU when standard LMWH or UFH doses are used. This is due to a heparin resistance with UFH or sub-optimal anti-Xa peak with LMWH that seems to be common in COVID-19 intensive care unit patients who received therapeutic anticoagulation. Consequently, it is important to measure anti-Xa levels for patients on therapeutic LMWH to ensure adequate dosing and continue with careful monitoring for those on UFH [86, 87].

### Is postoperative treatment for confirmed COVID-19 patients different?

#### Statement 8.1

COVID-19 surgical patient requires a multidisciplinary approach, above all if he/she is admitted in ICU for mechanical ventilation and presents with signs of septic shock (low level of evidence C).

#### Statement 8.2

Specific pharmacological treatment for COVID-19 disease is not available but when an empirical treatment is administered, it is mandatory to monitor for early detection of complications (moderate level of evidence B).

#### Statement 8.3

Currently, there are no data about the use of antimicrobial in COVID-19 patients to prevent secondary healthcare infections (low level of evidence C).

#### Statement 8.4

Initial prompt antibiotic therapy for intra-abdominal infections in surgical patients is typically empirical and depends on the underlying severity of infection, the pathogens presumed to be involved and the risk factors indicative of major resistance patterns. Antimicrobial treatment should be targeted to results from cultures from the site of infections or hemocultures with de-escalation of treatment as early as possible, in accordance with WSES guidelines (moderate level of evidence B).

#### Statement 8.5

Empirical anti-fungal treatment should only be considered in critically COVID-19 patients, presenting fever of unknown origin, with new pulmonary infiltrate superimposed on a viral pneumonitis pattern, with the aim of confirming the diagnosis by invasive techniques and/or the use of fungal biomarkers (moderate level of evidence B).

#### Recommendations 8

We recommend carefully administering antibiotics in COVID-19 surgical patients for the high risk of selecting resistant bacteria, especially in patients admitted in ICU for mechanical ventilation. Early empirical antibiotic treatment should be targeted to results from cultures, with de-escalation of treatment as soon as possible (strong recommendation based on the moderate level of evidence 1B).

We recommend against empirical anti-fungal treatment in all surgical COVID-19 patients but we suggest considering it in critically ill patients (strong recommendation based on the moderate level of evidence 1B).

#### Summary of evidence and discussion

COVID-19 patients who undergone emergency surgery can be divided in:

- Patients admitted to hospital for treatment of mild/severe COVID-19 infection who require ventilatory support and ICU hospitalization; they may develop an intra-abdominal disease needing for emergency surgeon’s evaluation and managing. An Italian epidemiological study showed that critically ill patients with laboratory-confirmed COVID-19 admitted to ICUs are above all older men (*n* = 786; age ≥ 64 years) and that the majority of these patients requires mechanical ventilation and high levels of positive end-expiratory pressure (PEEP). The mortality rate for this group of patients is 26% in this study [88].

Therefore, this group of critically ill patients is associated with a high risk of perioperative mortality and demands a multidisciplinary approach and a trained postoperative management (anesthesiologist, infection disease physician, surgeon, cardiologist, nephrologist).

- Patients admitted to the hospital with acute surgical pathologies, requiring surgeon’s evaluation and managing. After diagnostic ED work-up (RT-PCR, chest and abdominal CT or abdominal CT and CXR or lungs US), they could be divided in confirmed COVID-19 patients, suspected/uncertain for SARS-CoV-2 infection without confirmed diagnosis patients, and negative patients.

The group of suspected/uncertain patients needs to be isolated until RT-PCR swab test result is available, according to intra-hospital infection control policy.

Most common known COVID-19 infection complications are ARDS, arrhythmia, shock, acute cardiac injury, secondary infection, acute kidney injury and death which may occur in severe cases.

Currently, the data on the clinical characteristics and outcomes of patients with COVID-19 infection undergoing surgeries are rare.

Lei et al. [89] retrospectively analyzed data about 34 patients who were unintentionally scheduled for elective surgeries during the incubation period of COVID-19 to assess the impact of surgery on the outcomes of this group of patients given that surgery may cause an immediate impairment of cell-mediated immunity, one of the major mechanisms that bring viral infections under control.

Of the 34 operative patients, the median age was 55 years (IQR, 43–63), and 20 (58.8%) patients were women. All patients developed COVID-19 pneumonia shortly after surgery with abnormal findings on chest CT scans. Common symptoms included fever (31 [91.2%]), fatigue (25 [73.5%]) and dry cough (18 [52.9%]). Fifteen (44.1%) patients required admission to ICU during disease progression, and 7 patients (20.5%) died after admission to ICU. Compared with patients not requiring ICU admission, ICU patients were older, more likely to have underlying comorbidities, underwent more difficult surgeries, and more severe laboratory abnormalities (e.g. hyperleukocytemia, lymphopenia). The most common complications in non-survivors patients included ARDS, shock, arrhythmia and acute cardiac injury.

This retrospective study showed that surgery can have a negative impact on COVID-19 patients even if they are asymptomatic, with a mortality rate of 20.5% and the potentially needing for postoperative ICU care [89].

Another retrospective study carried out in a single thoracic department, enrolling 25 COVID-19 patients reported similar data showing that COVID-19 is associated with poor prognosis for patients undergoing surgery, especially for those with chronic diseases [90].

According to available data, coronavirus infection appears to be a complex systemic disease involving different organs. This lays on that angiotensin-converting enzyme 2 (ACE2) is the functional receptor for SARS-CoV-2.

Hammings et al. investigated the immuno-localization of ACE2 protein and reported that it is abundantly present in humans in the epithelia of the lung and small intestine including the duodenum, jejunum and ileum, but not in the colon. Furthermore, ACE2 is present in arterial and venous endothelial cells and arterial smooth muscle cells of various human organs such as oral and nasal mucosa, nasopharynx, lung, stomach, small intestine, colon, skin, lymph nodes, thymus, bone marrow, spleen, liver, kidney and brain. Moreover, ACE2 mRNA was found in many tissues and it is highly expressed in renal, cardiovascular and gastrointestinal tissues.

The binding of SARS-CoV-2 on ACE2 causes an elevated expression of ACE2, which can lead to damages primarily on alveolar cells and lately on other organs. Damages to alveolar cells can, in turn, trigger a series of systemic reactions with the secretion of various pro-inflammatory mediators that may play important roles in the pathophysiology of complications [51].

Several studies reported higher in-hospital mortality in patients with COVID-19 infection presenting abnormal kidney function, diabetes and cardiac injury [91–95].

Therefore, COVID-19 patients presenting already with comorbidities such as diabetes, renal abnormalities (e.g. haematuria, proteinuria, acute kidney injury), chronic obstructive pulmonary disease (COPD) and chronic cardiovascular disorders are at risk for presenting severe coronavirus disease as well as poor outcome after surgery.

Moreover, the duration of the surgical procedure and general anaesthesia can increase the risk of postoperative infections.

Specific pharmacological treatment for COVID-19 is not available [96, 97].

In this lack, in the early phase of the pandemic, many centres decided to use empirical antiviral therapy with darunavir or lopinavir in combination with ritonavir and oseltamivir and hydroxychloroquine on the basis of early promising results in terms of survival in critical COVID-19 patients reported in several studies.

In an open-label clinical study that showed some limitations in the methodology, Gautret et al. [98] reported a significant decrease in viral load among patients diagnosed with SARS-CoV-2 and treated with hydroxychloroquine when compared with control patients. In addition, they describe a synergistic effect in a subset of patients who received hydroxychloroquine with adjunctive azithromycin.

Azithromycin is a broad-spectrum macrolide antibiotic with a long half-life and a large volume of distribution; it is primarily used for the treatment of respiratory, enteric and genitourinary bacterial infections. In addition it has shown to be active in vitro against Zika and Ebola viruses [99] and to prevent severe respiratory tract co-infections when administered to patients suffering viral infection [95, 96]. It demonstrated also to have an anti-inflammatory activity that could mitigate the “cytokine storm” and organ damage in COVID-19 patients [100].

.In the early phase of this pandemic, lopinavir/ritonavir were administered in severe coronavirus disease cases, in association with other medical treatment. Cao et al. [101] conducted a randomized, controlled, open-label trial involving 199 hospitalized adult patients with confirmed SARS-CoV-2 infection and demonstrated that treatment with lopinavir–ritonavir is not associated with a difference from standard care in the time to clinical improvement. Instead, adverse effects observed include gastrointestinal distress such as nausea, diarrhoea and hepatotoxicity. Moreover, particular attention needs to be focused on pharmacokinetics interactions involving inhibition of CYP3A4 and some transporters, inducted by antiviral treatments.

While in healthy, young patients diagnosed with COVID-19 the combination of these drugs are well tolerated with mild side effects, in the elderly patients presenting age-related comorbidities that result in complex polypharmacy, the risk of toxicity with lethal complications is increased.

Furthermore, physiological changes related to ageing may affect both pharmacokinetics and pharmacodynamics thereby putting elderly patients at risk of inappropriate prescribing and adverse drug reactions [102].

Both lopinavir/ritonavir and chloroquine and hydroxychloroquine are known to cause QT prolongation and torsades de pointes. In particular, long-term chloroquine and hydroxychloroquine use may increase depolarization length duration and Purkinje fiber refractory period, ultimately leading to atrioventricular nodal and/or His system malfunction and arrhythmia. QT prolongation in individual medical therapy is not always predictable; dose adjustments and/or additional monitoring with electrocardiograms may be appropriate in some cases [103].

COVID-19 patients treated with these drugs need a careful clinical and biological multidisciplinary surveillance.

Currently, there are no data about the use of antimicrobial in COVID-19 patients.

Several studies [104, 105] reported that secondary infections following respiratory viral illnesses are common, most frequently involving the lower respiratory tract, with *Streptococcus pneumoniae*, *Haemophilus influenzae* and *Staphylococcus aureus* being the most frequently reported pathogens. The exact incidence of bacterial superinfection in COVID-19 is unknown. It was reported that the most common co-infections in COVID-19 patients were rhinovirus/enterovirus (6.9%), respiratory syncytial virus (5.2%) and non–SARS-CoV-2 Coronaviridae (4.3%), isolated in the respiratory tract [105].

Consequently, taking into consideration the risk potential for co-infections of the respiratory tract, confirmed COVID-19 patients were often administered with antibiotics even if there is no any confirmation of the co-infections. This could contribute in selecting microbes, increasing the alarming problem of antimicrobial resistance, above all in critical ill patients, in mechanical ventilatory support.

If antimicrobial treatment is considered, a beta-lactam providing coverage for *S. pneumoniae* ± methicillin-susceptible *S. aureus* should be the first option (e.g. amoxicillin + clavulanic acid or third-generation cephalosporins). Once-a-day administration (where applicable) or continuous administration of beta-lactam antibiotics should be considered to decrease the use of personal protective equipment which may be in short supply in many places. Macrolides and quinolones should be avoided because of their cardiac side effects, considering that other agents associated with cardiac side effects such as hydroxychloroquine and lopinavir/ritonavir are used in many places notwithstanding the limited evidence for their efficacy and impact on antimicrobial resistance [106]. If atypical coverage is considered necessary (e.g. COVID-19 not yet confirmed and suspicion of Legionella infection), consideration should be given to doxycycline. For patients in intensive care units requiring mechanical ventilation, standard measures to prevent ventilator associated pneumonia and other healthcare-associated infections should be applied according to local and individual patient-level resistance. Empirical treatment should always be adapted to microbiological results [106].

The administration of antibiotics in surgical confirmed COVID-19 patients, except azythromicin used in many empirical protocols but not still validated, should follow WSES recommendations for the management of intra-abdominal infections [107]. Initial prompt antibiotic therapy for intra-abdominal infections (IAIs) in surgical patients is typically empirical and depends on the underlying severity of infection, the pathogens presumed to be involved and the risk factors indicative of major resistance patterns. Antimicrobial treatment should be targeted to results from cultures from the site of infections or hemocultures with de-escalation of treatment as early as possible. To ensure timely and effective administration of antimicrobial therapy for critically ill patients, clinicians must consider the pathophysiological and immunological status of the patient as well as the pharmacokinetic properties of the employed antibiotics. In fact, COVID-19 patients could present with renal failure and modification in liver function.

If there are no signs of persistent leukocytosis or fever, antimicrobial therapy for intra-abdominal infections should be shortened for patients demonstrating a positive response to treatment.

The risk of fungal co-infection appears to be low [108], despite the fact that viral infections such as influenza and a large number of classical risk factors (e.g. haematological patients, solid-organ and haematopoietic stem cell transplant recipients, HIV patients) have been described as a favourable environment for invasive fungal infections, especially for invasive pulmonary aspergillosis [109]. Empirical anti-fungal treatment should only be considered in critically patients with new pulmonary infiltrate superimposed on a viral pneumonitis pattern, with the aim of confirming the diagnosis by invasive techniques and/or the use of fungal biomarkers [110].

Furthermore, in severe SARS-CoV-2 pneumonia, high dose of corticosteroids are administered. The rationale for the use of corticosteroids is to decrease the host inflammatory responses in the lungs, which may lead to acute lung injury and ARDS. However, this benefit may be outweighed by adverse effects, including delayed viral clearance and increased risk of secondary infection. In patients taking corticosteroids (long-term use of corticosteroids or perioperative use of corticosteroids), the risk for anastomotic leakage is significantly increased, such as wound infection and wound dehiscence. It is known that corticosteroids impair wound healing by decreasing activation and infiltration of inflammatory cells. These inflammatory cells, macrophages and polymorph leucocytes, are essential in the first phase of wound healing. Additionally, corticosteroids inhibit the expression of growth factors and matrix proteins such as collagen synthesis. Other known complications of glucocorticoids include gastrointestinal bleeding, peptic ulcer perforation and sigmoid diverticular perforation [111]. The clinical outcomes of coronavirus and similar outbreaks do not support the use of corticosteroids. In a retrospective observational study of 309 adults who were critically ill with MERS, patients who were given corticosteroids were more likely to require mechanical ventilation, vasopressors and renal replacement therapy [112]. For the management of SARS, corticosteroid treatment was more associated with psychosis, diabetes and avascular necrosis [113, 114]. Overall, there is no unique reason to expect that patients with COVID-19 infection will benefit from corticosteroids, and such treatment may be harmful [115]. The potential harms and lack of proven benefit for corticosteroids cautions against their routine use in patients with COVID-19 outside an RCT, unless a concomitant compelling indication, such as chronic obstructive pulmonary disease exacerbation or refractory shock, exists. Therefore, glucocorticosteroids should not be given to patients routinely but in very selected cases. Recently, study showed that in patients hospitalized with COVID-19, the use of dexamethasone resulted in lower 28-day mortality among those who were receiving either invasive mechanical ventilation or oxygen alone at randomization but not among those receiving no respiratory support (https://www.nejm.org/doi/10.1056/NEJMoa2021436).

In the management of postoperative pain in COVID-19 patients, concerns were expressed about the administration of non-steroidal anti-inflammatory drugs (NSAIDs). Their use has been associated with gastrointestinal complications and cardiovascular and renal adverse effects. According to low-quality data, NSAIDs (ibuprofen/ketoprofen) have reported to play a role in decreasing host defence with serious infectious complications. In the lack of scientific evidences to establish a correlation between NSAIDS and the worsening of COVID-19 pneumonia, patients should be advised against any NSAID self-medication when COVID-19-like symptoms begins. NSAIDs should be used with extreme caution [116].

### Is it necessary to create an overt COVID-19 patients surgical ward?

#### Statement 9.1

Patients needing for a surgical procedure or who have undergone urgent surgery with confirmed SARS-CoV-2 infection require a multidisciplinary approach and management, and they need to be isolated from negative patients to decrease the in-hospital risk of virus transmission and environmental contamination (very low level of evidence D).

#### Statement 9.2

Confirmed COVID-19 patients need to be cared by a trained and skilled workforce with adequate PPEs (e.g. N95 masks, goggles, double gloves, face mask and protective gowns) to preserve negative surgical patients from contagion, because of the high risk to come in contact with droplets and biological fluids (very low level of evidence D).

#### Recommendations 9

After an emergency surgical procedure, we recommend re-admitting in COVID ICU patients with severe pneumonia for management and monitoring.

For stable asymptomatic or mild symptomatic COVID-19 patients, it would be better to create a surgical dedicated ward with the aim to avoid any contamination of negative patients and to limit the in-hospital exposure to the virus to a dedicated and trained team (strong recommendation based on very-low-quality evidence 1D).

#### Summary of evidence and discussion

Since the outbreak of COVID-19 pandemic, in the majority of hospitals, medical and surgical wards were converted to admit infected patients, because the tremendous amount of patients requiring hospital admission and ventilatory support.

In the early phase of the pandemic, to assure the management of infected and non infected surgical patients, hospitals were divided in dedicated HUB for overt COVID-19 patients, with limited surgical staff and ORs, for those infected patients requiring surgery, and COVID-free facilities for elective, emergency surgery and urgent oncological procedures in negative COVID-19 patients, with the aim of minimizing the risk of exposure to the virus and for better access and use of limited resources and PPE, but the progression of contagion has not allowed this for long time.

Moreover, in the early phase of the COVID-19 surge, the availability of RT-PCR test was very limited and asymptomatic patients admitted in the ED were not tested for COVID-19 routinely, and this has contributed to the nosocomial spreading of the virus. The outcome of this practice was an increasing number of infected physicians and HCWs, warranting aggressive measures of protection (such as N95 masks, goggles or face mask and protective gowns) to ensure safety during this COVID-19 outbreak, which are often lacking.

Current evidence suggest that even non-symptomatic infected patients can spread COVID-19 disease with high efficiency. This has implied to reconsider the indication of performing test for COVID-19 in all surgical patients to limit the contagion to HCWs and the in-hospital spreading of the virus [5].

In clinical practice, patients needing for a surgical procedure or undergone urgent surgery with confirmed SARS-CoV-2 infection require a multidisciplinary approach and management, and they need to be isolated from other negative surgical patients to decrease the in-hospital risk of virus transmission and environmental contamination.

It is established that :

- 2019-nCoV can be transmitted by asymptomatic infectors;
- 2019-nCoV is transmitted by droplets, fomites and closed contact;
- Faecal-oral and aerosol transmission is involved in the spreading of COVID-19 disease;
- Human coronaviruses can persist on inanimate surfaces such as metal, glass or plastic for up to 9 days.

On this basis, confirmed COVID-19 patients need to be cared by a trained and skilled workforce with adequate PPEs (e.g. N95 masks, goggles, double gloves, face mask and protective gowns) to preserve negative surgical patients from contagion, because of the high risk to come in contact with droplets and biological fluids.

After emergency surgery, patients with severe COVID-19 pneumonia need to be re-admitted in COVID ICU for management and monitoring

For stable asymptomatic or mild symptomatic COVID-19 patients, it would be better to create a surgical dedicated ward with the aim to avoid any contamination of negative patients, patients admitted for acute abdomen and patients waiting for a surgical procedure and to limit the in-hospital exposure to the virus to a dedicated and trained team.

In the postoperative time, an overt COVID-19 patient who had undergone surgery could present with an ileostomy or a colostomy, with a nasogastric tube or intra-abdominal drainages to manage with risk of healthcare contamination and dissemination of the viral disease. Furthermore, asymptomatic or pauci-symptomatic COVID-19 patients could present with a rapid progression of the infection to be promptly recognized, requiring ventilatory support and adequate monitoring.

Patients selected for NOM have to be closely evaluated by the surgeon to decide for surgery if it is necessary.

In the postoperative period, it is very important to carefully administer empirical treatment for COVID-19 infection when it is indicated, antibiotics and other drugs to avoid side effects, sometimes lethal. In selected patients, principles of fast-track surgery [115], such as optimum analgesia, reduction of postoperative nausea and vomiting, and early ambulation and feeding, might be applied to decrease the length of hospital stay and the need of available beds for positive patients requiring to be admitted in a surgical ward.

### Is it necessary to create a suspected COVID-19 patients surgical ward?

#### Statement 10.1

Insufficient precaution in managing a false-negative COVID-19 patient could cause the contagion to nurses, surgeons and negative patients (QoE moderate B).

#### Statement 10.2

A positive RT-PCR test for COVID-19 test has more weight than a negative test because of the test’s high specificity but moderate sensitivity (QoE moderate B).

#### Statement 10.3

A single negative COVID-19 test should not be used as a rule-out in patients with strongly suggestive symptoms (QoE low C)

#### Recommendations 10

Considering the high infectivity related to SARS-CoV-2, we recommend isolating suspected/uncertain patients to ensure the limiting of in-hospital exposure and contagion (strong recommendation based on the low level of evidence 1C).

If suspected/uncertain COVID-19 patient needs to undergo immediate surgery, we recommend managing him/her like a confirmed COVID-19 patient and isolating the patient after surgery, until diagnosis is confirmed by the RT-PCR test, which has to be performed twice in uncertain patients (patients with strongly suggestive symptoms, radiological features for COVID-19 disease and negative RT-PCR test). If the RT-PCR swab test is negative, but CT scan showed signs of COVID-19 pneumonia, the patient cannot be considered as COVID-19 (–), and he/she has to be isolated and the RT-PCR swab test has to be repeated after 48–72 hours; if the RT-PCR swab test is positive, the patient is considered COVID-19 (+) and addressed to the COVID ICU or COVID surgical unit (strong recommendation based on the low level of evidence 1C).

#### Summary of evidence and discussion

Suspected or uncertain COVID-19 patients, those patients with RT-PCR swab test and thoracic radiological imaging result discrepancy, with abdominal pathology requiring urgent surgery could represent a potential cause of nosocomial spread of viral disease. Disease-free patients can be transferred to the regular surgical ward for their management. There is no evidence about the management of uncertain/suspected COVID-19 patients, presenting risk factors to present COVID-19 disease.

Every patient undergoing surgery should be screened by the RT-PCR test for SARS-CoV-2 infection before entering the operating room, even if he is asymptomatic, and he/she should be treated at first such as an infected patient, until suspected diagnosis is confirmed.

A nasopharyngeal swab is usually the collection method used to obtain a specimen for testing. Nasopharyngeal specimens may miss some infections, and a deeper specimen may need to be obtained by bronchoscopy, which is a high-risk AGP. Alternatively, repeated testing can be used because over time, the likelihood of the SARS-CoV-2 being present in the nasopharynx increases.

The use of serology to facilitate the diagnosis of SARS-CoV-2 infection when a nasopharyngeal swab specimen was collected inappropriately and the molecular assays were performed unsatisfactorily, can be taken in consideration, if it is available.

The incubation period for SARS-CoV-2 reaches up to 14 days with a mean duration of 5.2 days; consequently, an initial negative result of the pharyngeal swab can turn into a positive result few days later [6, 7]. Furthermore, the concomitance of an intra-abdominal infection can delay the real understanding of the clinical features.

Interpreting the result of a test for COVID-19 depends on two things: the accuracy of the test, and the pretest probability or estimated risk of disease before testing.

A positive RT-PCR test for COVID-19 test has more weight than a negative test because of the test’s high specificity but moderate sensitivity.

A single negative COVID-19 test should not be used as a rule-out in patients with strongly suggestive symptoms [117].

Considering the high infectivity related to SARS-CoV-2, insufficient precaution in managing a false-negative patient could cause the contagion to nurses and surgeons. Consequently, if the suspected/uncertain COVID-19 patient needs to undergo immediate surgery, he has to be managed like a confirmed COVID-19; alternatively, the suspected/uncertain patient should be isolated to ensure the limiting of exposure and contagion.

After surgery, if diagnosis is not yet confirmed or eliminated, the patient has to be kept in isolation in a single room with a negative pressure, until RT-PCR test, or serology, result is available.

Postoperative rounds, medications and wound management should be performed under personal protection to avoid contact with secretions.

Since surgical stress could increase the viral load and make the healthy carrier more infectious; daily assessment of body temperature as well as respiratory symptoms is mandatory for this group of patients: all postoperative fevers should be kept in serious consideration such as expression of SARS-CoV-2 infection, and the pharyngeal swab test should be repeated several times unless a clinical explanation of the ongoing high temperature would be found.

If patient was selected for NOM, he has to be isolated from the other negative surgical patients, for the same reasons explained above since infection is confirmed or eliminated.

As soon as the patient is recovered, think to discharge him from hospital.

### Are there different discharge policies for suspected/overt COVID patients?

#### Statement 11.1

Current data reported that several patients meeting the criteria for hospital discharge could show positive RT-PCR test after the established quarantine of 14 days (low level of evidence C).

#### Statement 11.2

There is no data proving the contagiosity of a recovered patient who has been kept intermittently to eliminate SARS-CoV-2 after 14 days from the onset of symptoms or positive RT-PCR test.

#### Recommendations 11

We suggest that after hospital discharge, all the confirmed asymptomatic surgical COVID-19 patients should be kept in isolation for at least 2 weeks have passed since their first positive RT-PCR nasopharyngeal swab test (weak recommendation based on very low quality of evidence 2D).

We recommend obtaining a negative RT-PCR nasopharyngeal swab test before discontinuing isolation and precautions earlier than 14 days after the confirmed diagnosis of COVID-19 (strong recommendation based on very low level of evidence 1D).

We recommend evaluating immunocompromised patients’ discharge in a multidisciplinary approach (strong recommendation based on very low level of evidence 1D)

#### Summary of evidence and discussion

The discharge from hospital of uncertain/suspected or confirmed, asymptomatic or mild symptomatic cases of COVID-19—if clinically appropriate, after recovery from the intra-abdominal infection—may be considered as soon as possible.

According to WHO recommendations, the clinical criteria for hospital discharge of a confirmed COVID-19 patient are [3]:

- Normal temperature lasting longer than 3 days without the use of fever-reducing medications
- Significantly relieved respiratory symptoms, if patient was symptomatic
- Substantially improved acute exudative lesions on chest CT
- A series of two repetitive negative RT-PCR test results with at least 1-day interval.

According to the CDC recommendations [https://www.cdc.gov/coronavirus/2019-ncov/hcp/disposition-hospitalized-patients.html], the patient could be discharged to home if:

- Appropriate caregivers are available at home, in case of isolated elderly patients
- The patient can be isolated limiting the risk of exposure for the other household members (e.g. single room with good ventilation, face mask wear, reduced close contact with family members, separate meals, good hand sanitation, no outdoor activities)
- There are no household members who may be at increased risk of complications from COVID-19 infection (e.g. people > 65 years old, young children, pregnant women, people who are immunocompromised or who have chronic heart, lung or kidney conditions)
- All the household members are capable of adhering to recommended precautions to avoid the shedding of the virus until the risk of transmission is low.

The decision to remove precautions is based on two general strategies: a test-based strategy that requires negative respiratory RT-PCR tests obtained after the resolution of symptoms and a symptom-based strategy that recommends keeping patients on contact precautions until a fixed period has elapsed from symptom recovery. The underlying assumption of the symptom-based strategy is that waiting for a fixed period of time is a surrogate for negative RT-PCR testing, which itself is a surrogate for the absence of shedding infectious virus.

After hospital discharge, if there is a possibility that the patient is still infectious, clear advice should be given on how to avoid transmitting COVID-19 disease and all the confirmed and suspected COVID-19 patients should be further quarantined in designated COVID (+) hospitals, healthcare facilities or at home, keeping precautions to avoid infection transmission, followed up by RT-PCR tests, to decide to discontinue the isolation, even if the recovered is asymptomatic. COVID-19 patients should be kept in isolation at least until the 14 days have passed since the date of their first positive COVID-19 diagnostic test, as clinical criteria cannot be used to assess where these individuals are in the course of their illness [3–121].

Due to the convenience of operation and the acceptability of patients, the most commonly used specimens at discharge in practice are also oropharyngeal swabs, and sometimes, nasal swabs are collected at the same time. However, in the middle and later stages of the disease, the amount of virus remaining in the pharyngeal cells is small or very low in some patients and they can result (false) negative. If only the pharyngeal specimens are taken, the viral nucleic acid cannot be detected. Although alveolar lavage fluid is easier to detect viruses, due to its inconvenient operation and high risk of exposure, it is mainly used on critically ill patients who have been intubated [13]

This is consistent with prior literature [122], emphasizing that a single negative test does not rule out disease in patients with a high pretest probability of COVID-19. Repeated samples may improve yield. For example, among patients with a high pretest probability for COVID-19 and a negative nasopharyngeal swab, repeating the nasopharyngeal swab and also collecting a saliva sample may be considered [123].

It is important to note that with the recovery of the disease, the positive rate of oropharyngeal swabs in mild patients declines the fastest, and in the later course of the disease, positive results of anal swabs are more than that of pharyngeal swabs [13].

Furthermore, current data showed that several patients meeting criteria for hospital discharge could present positive RT-PCR test during the isolation period. The time from hospital discharge to positive checked RT-PCR after recovery was 5–13 days. All these patients continued to be asymptomatic and chest CT showed no changes from previous images [124–126].

These findings suggested that at least a proportion of recovered COVID-19 patients still may be virus carriers, that intermittent virus shedding might occur in recovered patients and that the number of PCR false-negative test patients at the discharge is not negligible.

Serology can help in detecting the infection in suspected/uncertain/asymptomatic cases and in deciding for discontinuation of quarantine. Antibodies IgM and IgG against SARS-CoV-2 infection are usually produced in infected patients, and antibody IgG can persist a very long time. There are no data about the use of serology to follow recovered patients.

An observational analysis of 150 patients and HCWs showed that the average time to transition from RT-PCR positive to negative was 24 days after symptom onset, and 10% of patients remained positive even 33 days after symptom onset [127].

Although there is no firm evidence indicating that these recovered patients would transmit the virus to others, after discharge, 4 weeks of further isolation with regular health monitoring (e.g. follow-up visits, phone calls, video-consultations) should be considered for COVID-19 patients, such as keeping all necessary precautions (face mask, reduced close contact) in order to protect family members and the community from infection and further spread of SARS-CoV-2.

## Conclusions

This position paper represents the result of an extensive analysis of the first consistent evidence about COVID-19 disease. The aim is to provide an evidence-based guidelines for emergency surgeons to perform safe surgery during this pandemic to limit the diffusion of the SARS-CoV-2 infection and to decrease mortality rate related to COVID-19 surgical patients. We recommend screening for COVID-19 infection at the emergency department all acute surgical patients who are waiting for hospital admission and urgent surgery. The screening work-up provides a RT-PCR nasopharyngeal swab test and a baseline (non-contrast) chest CT or a chest X-ray or a lungs US, depending on skills and availability. The management of COVID-19 surgical patients is multidisciplinary. If an immediate surgical procedure is mandatory, whether laparoscopic or via open approach, we recommend doing every effort to protect the operating room staff, for the safety of the patient.

## Data Availability

Not applicable

## Abbreviations

RT-PCR: Real-time quantitative polymerase chain reaction
CT: Computed tomography
US: Ultrasound
CXR: Chest X-ray
POCUS: Point-of-care ultrasound
ED: Emergency department
PPE: Personal protective equipment
ICU: Intensive care unit
TACS: Timing of Acute Care Surgery classification
OR: Operating room
ARDS: Acute respiratory distress syndrome
ECMO: Extracorporeal membrane oxygenation
COPD: Chronic obstructive pulmonary disease
PEEP: Positive end-expiratory pressure
HCW: Health care worker
LMWH: Low-molecular-weight heparin
AGP: Aerosol-generating procedures
IAI: Intra-abdominal infection
